# Supplementary material for: Symbiotic Microorganisms and Their Different Association Types in Aquatic and Semiaquatic Bugs
Source: Microbiol Spectr. 2022 Nov 21;10(6):e02794-22. doi: 10.1128/spectrum.02794-22 (PMC9769989; doi:10.1128/spectrum.02794-22)
Supplement: Supplemental file 1 — Fig. S1 to S22 and Tables S1 to S8. Download spectrum.02794-22-s0001.pdf, PDF file, 6.3 MB [file spectrum.02794-22-s0001.pdf]

Supplementary Figures

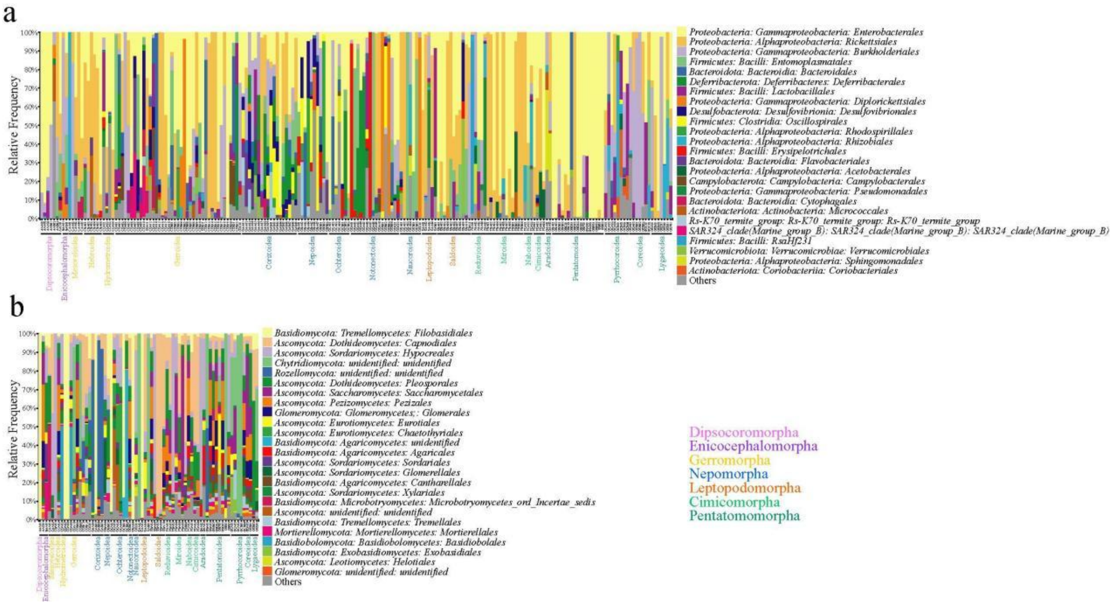

Fig. S1. Composition of symbiotic bacterial and fungal communities in true bugs.

Relative abundance plots of bacterial orders (a) and fungal orders (b) are shown, respectively. The infraorders or superfamilies are shown at the bottom of each plot, and are colored according to the infraorders or the infraorders to which they belong.

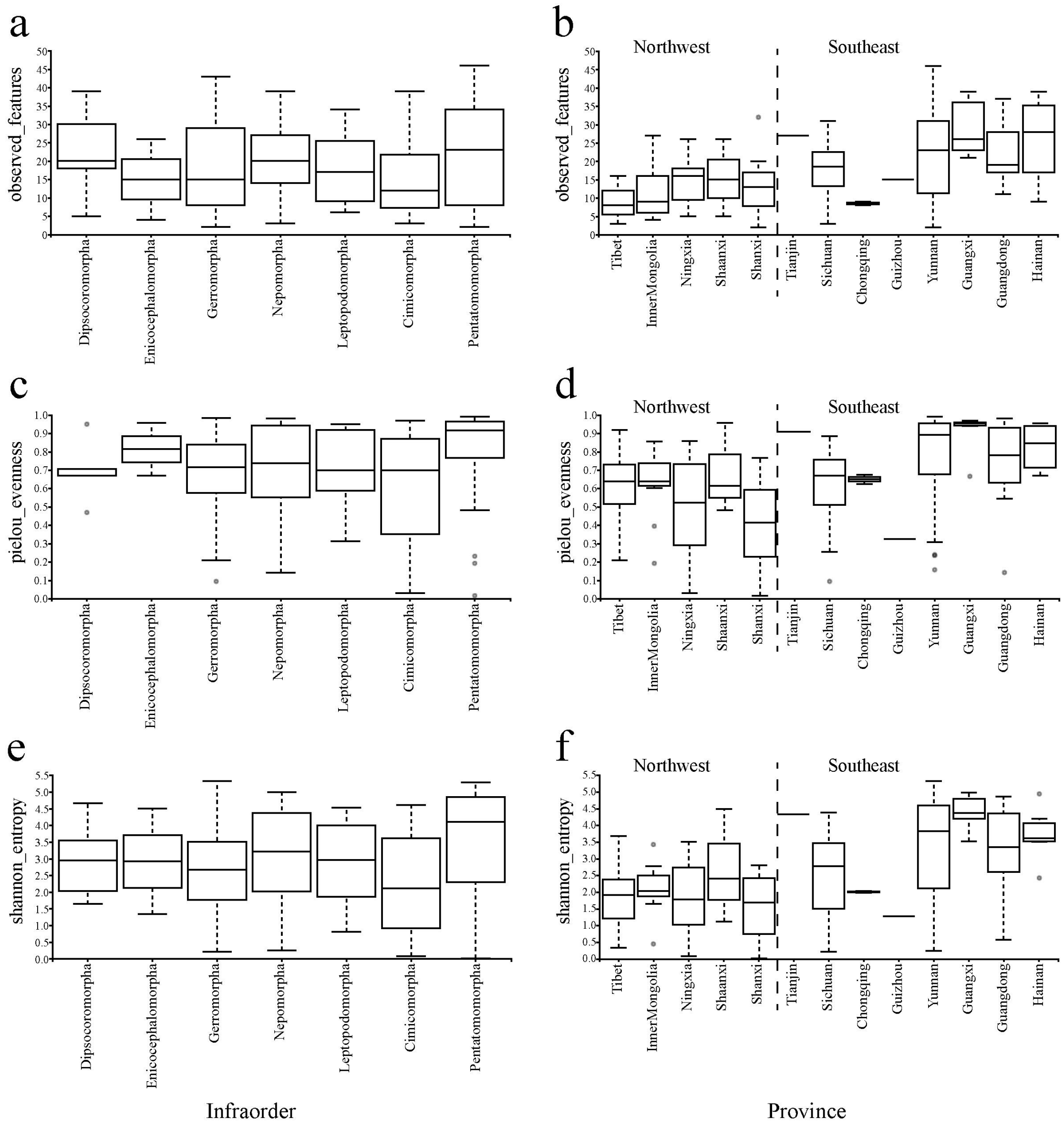

Fig. S2. The alpha diversity of symbiotic bacterial communities for samples in different infraorders and provinces. (a) (b) observed features, (c) (d) Pielou's evenness, and (e) (f) Shannon entropy indices of bacterial communities in seven infraorders and different provinces.

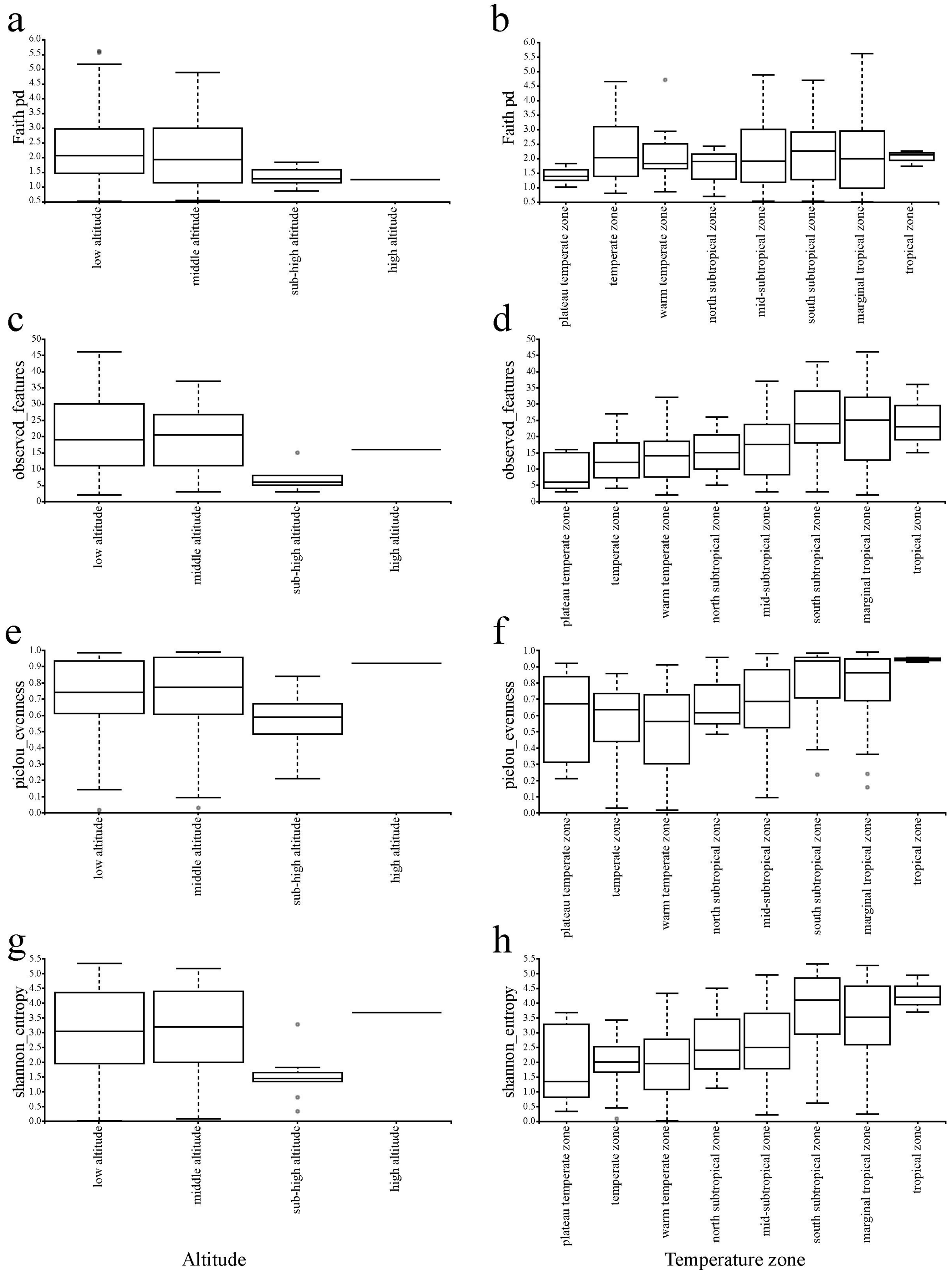

Fig. S3. The alpha diversity of symbiotic bacterial communities for samples in different altitudes and temperature zones. (a) (b) Faith's PD, (c) (d) observed features, (e) (f) Pielou's evenness, and (g) (h) Shannon entropy indices of bacterial communities in different altitudes and temperature zones.

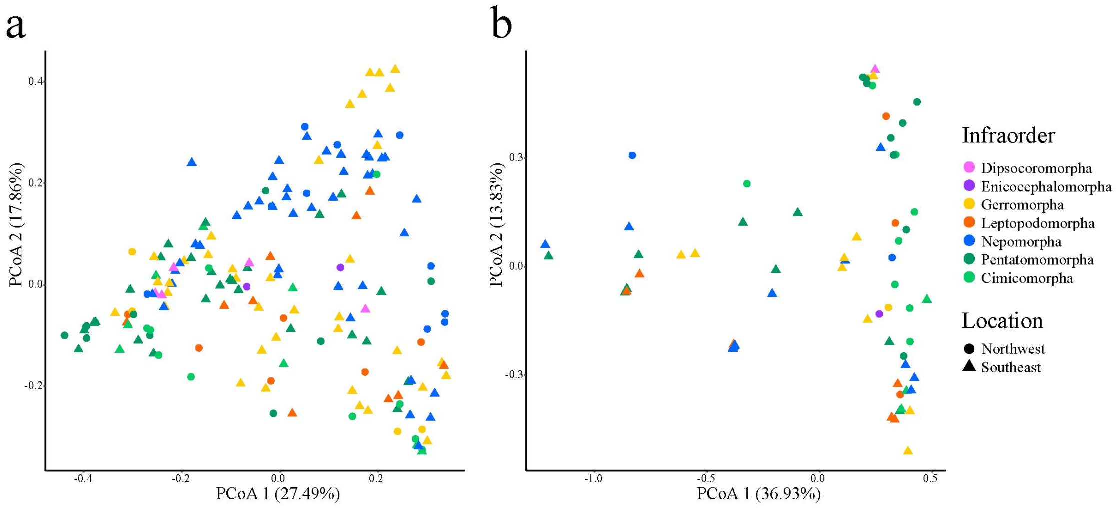

Fig. S4. PCoA plots of bacterial (a) and fungal (b) communities based on the weighted UniFrac distance.

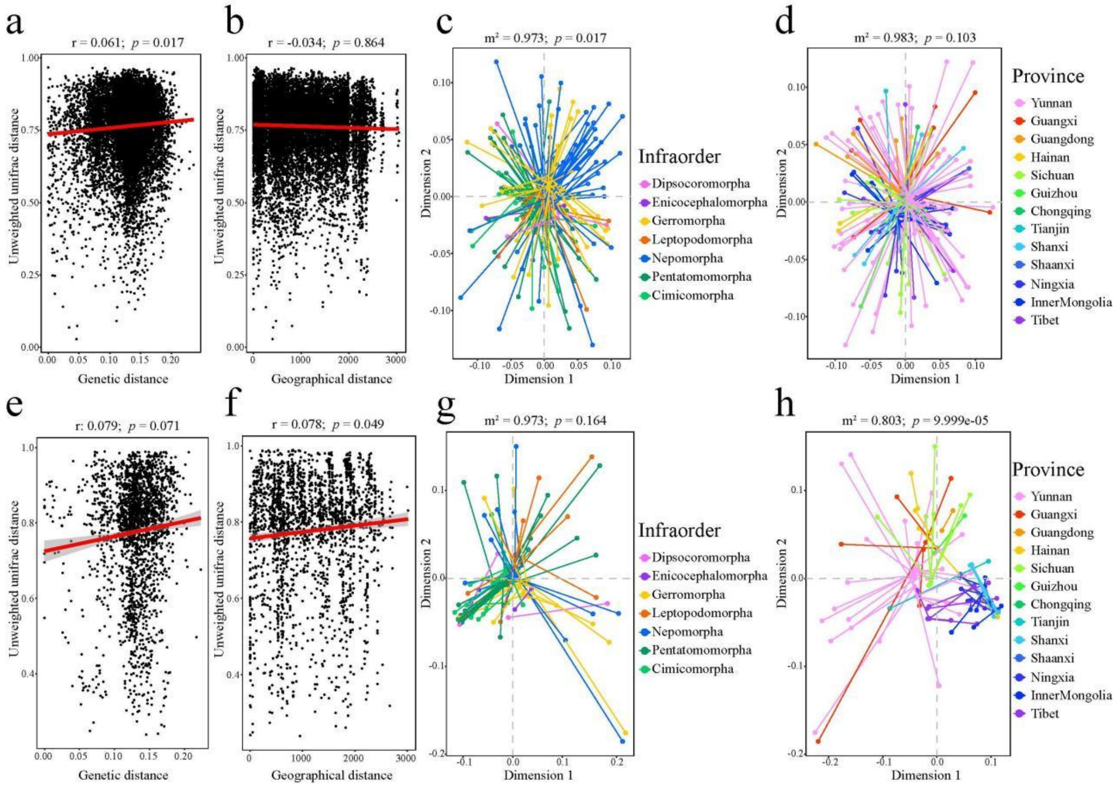

Fig. S5. The Mantel test and Procrustes test further confirm the effects of hosts on bacterial communities, and the effects of sample collecting area on fungal communities. a-d show the Mantel and Procrustes tests of bacterial communities. e-h

are the two tests of fungal communities. The genetic distance of host true bugs and the unweighted UniFrac distance of symbiotic microbial communities were used to test the impact of hosts to microbial communities. The geographical distance of collected sites and the unweighted UniFrac distance of symbiotic microbial communities were used to test the impact of environment on microbial communities. According to the Mantel test (a, b) and Procrustes test (c, d), the symbiotic bacterial communities are significantly affected by hosts (a, c) and not significantly influenced by environment (b, d). The symbiotic fungal communities are not significantly affected by hosts (e, g), and significantly influenced by collecting areas (f, h).

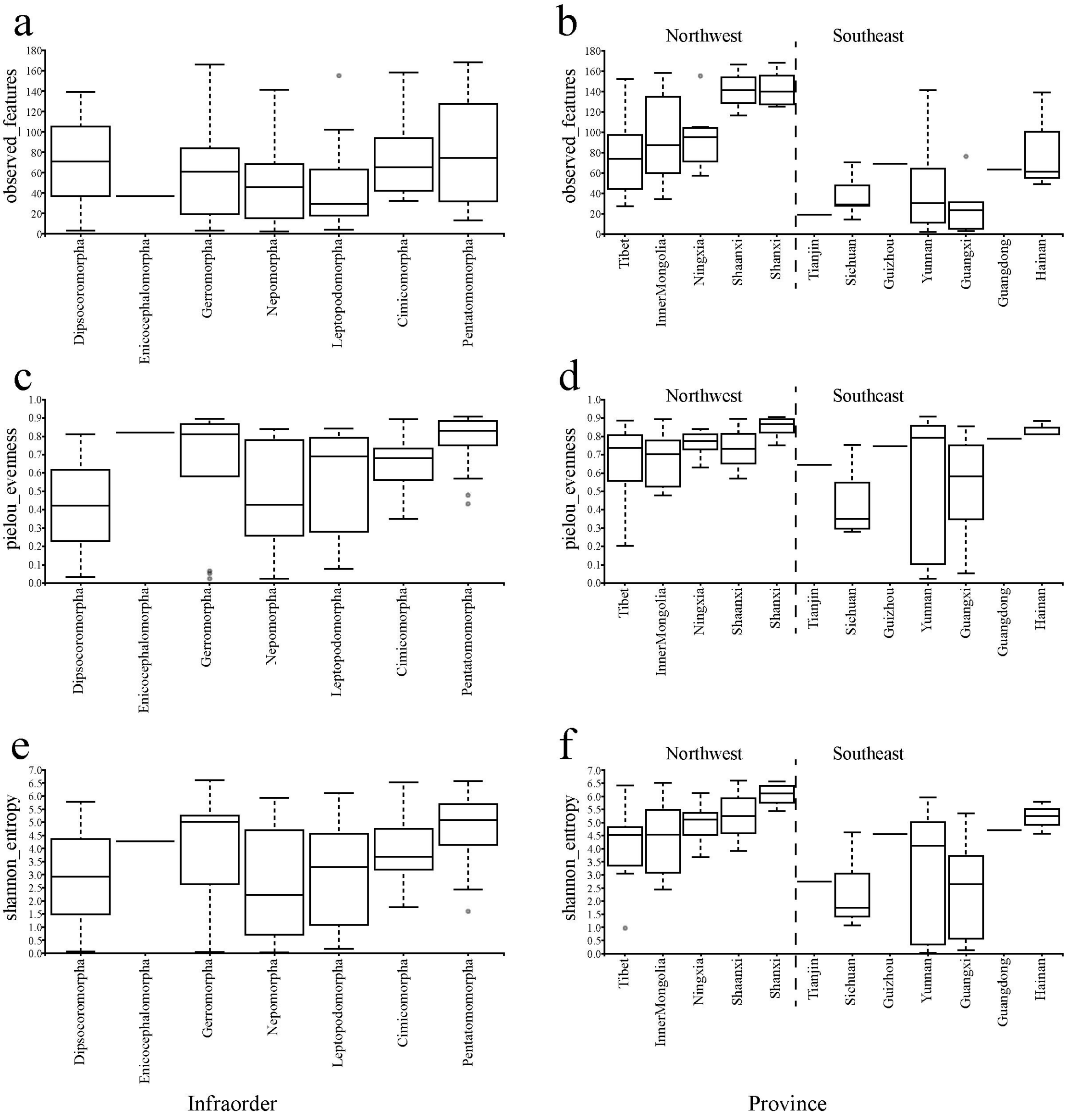

Fig. S6. The alpha diversity of symbiotic fungal communities for samples in different infraorders and provinces. (a) (b) observed features, (c) (d) Pielou's evenness, and (e) (f) Shannon entropy indices of fungal communities in seven infraorders and different provinces.

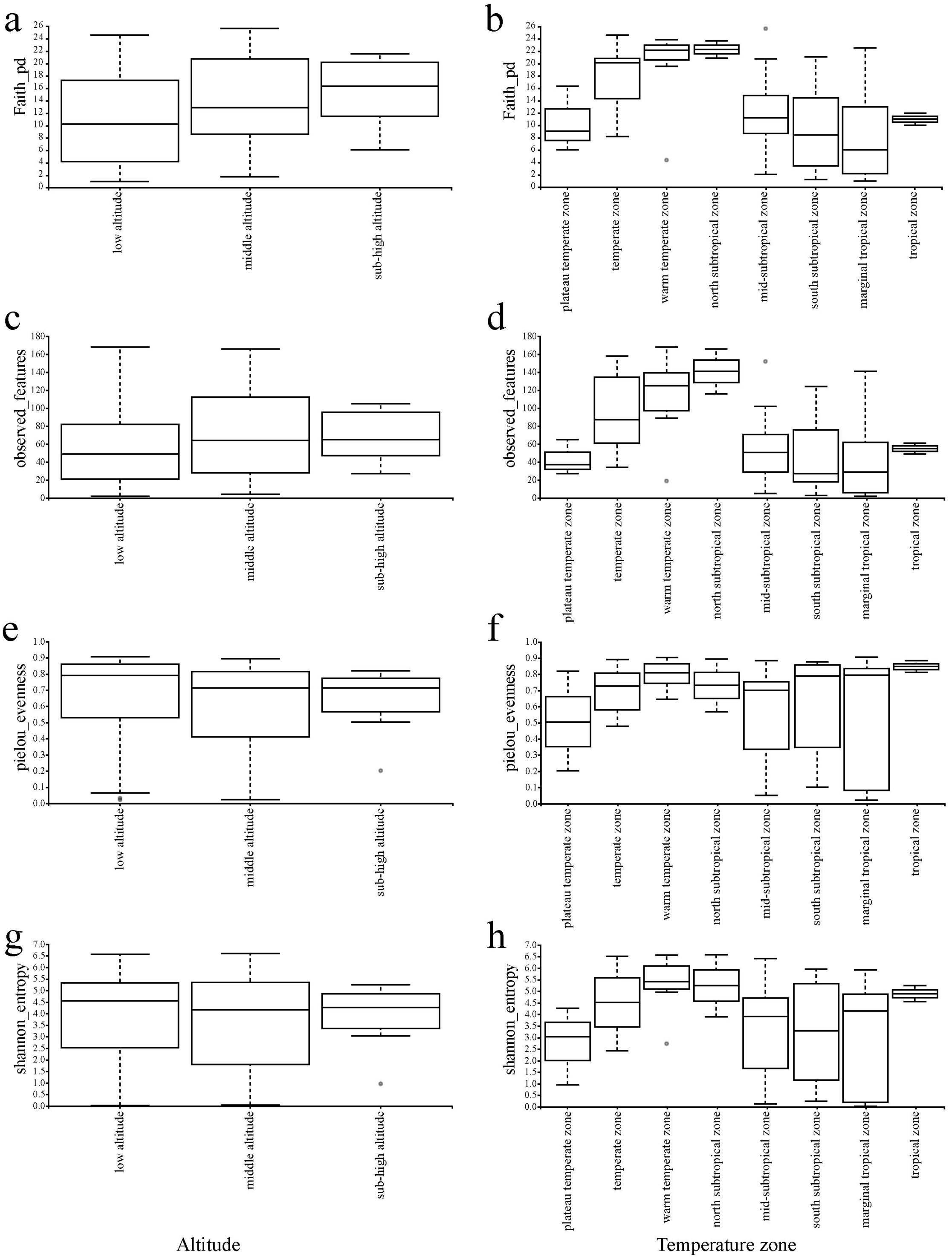

Fig. S7. The alpha diversity of symbiotic fungal communities for samples in different altitudes and temperature zones. (a) (b) Faith's PD, (c) (d) observed features, (e) (f) Pielou's evenness, and (g) (h) Shannon entropy indices of fungal communities in different altitudes and temperature zones.

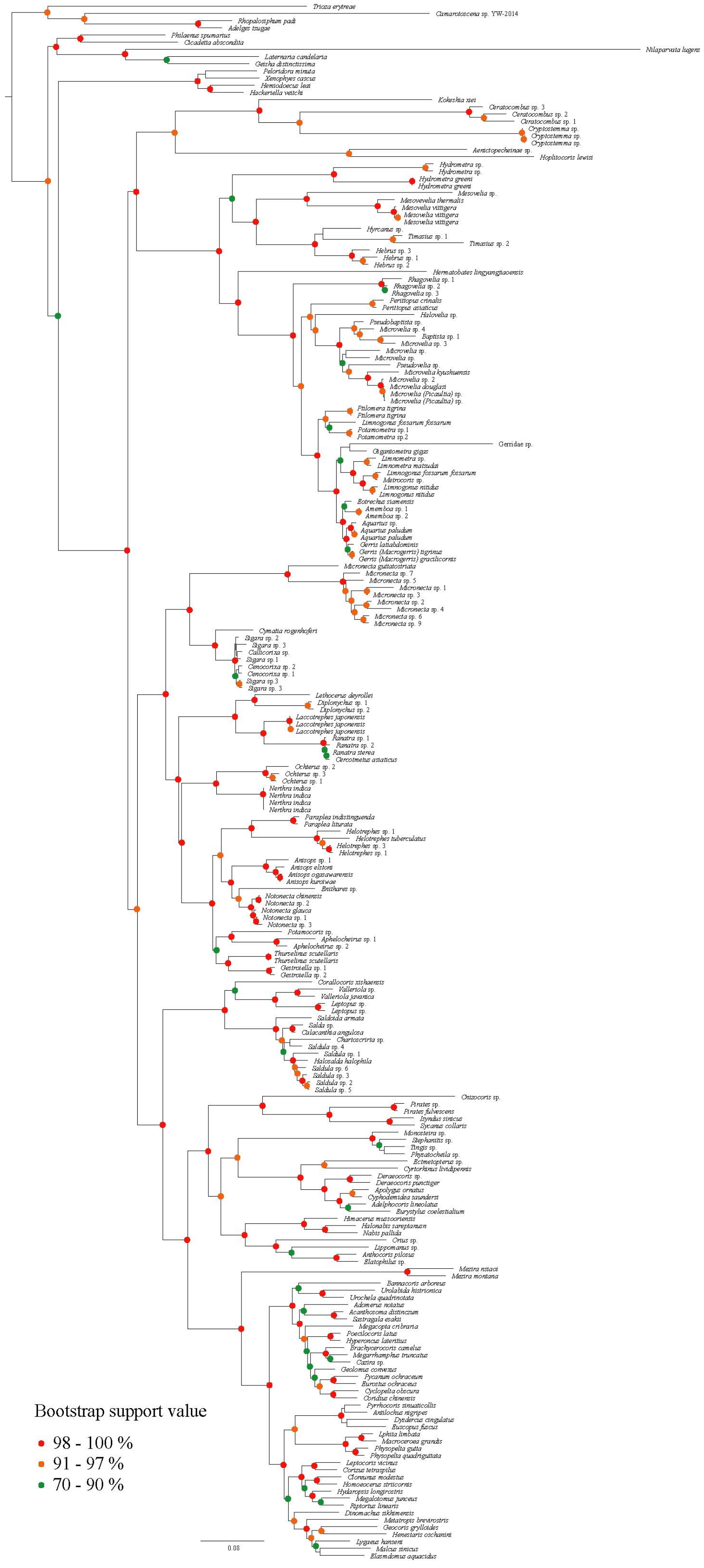

Fig. S8. Phylogenetic relationships of true bugs.

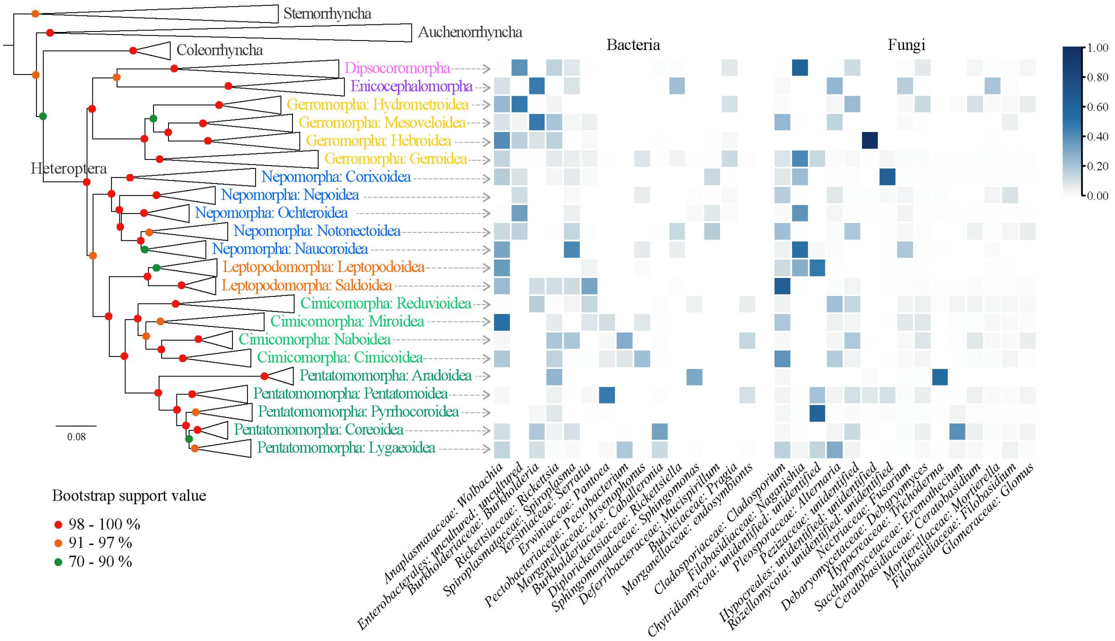

Fig. S9. The phylogenetic relationships of host true bugs and the corresponding heatmap of the abundance of symbiotic microbial communities at genus level. Branches were grouped to display the tree at infraorder and superfamily level. The superfamilies marked in the phylogenetic relationships are colored according to the infraorder to which they belong. The ML-tree with all branches was shown in the Figure S4. The heatmap on the left represents the 15 most abundant bacterial genus. The heatmap on the right represents the 15 most abundant fungal genus.

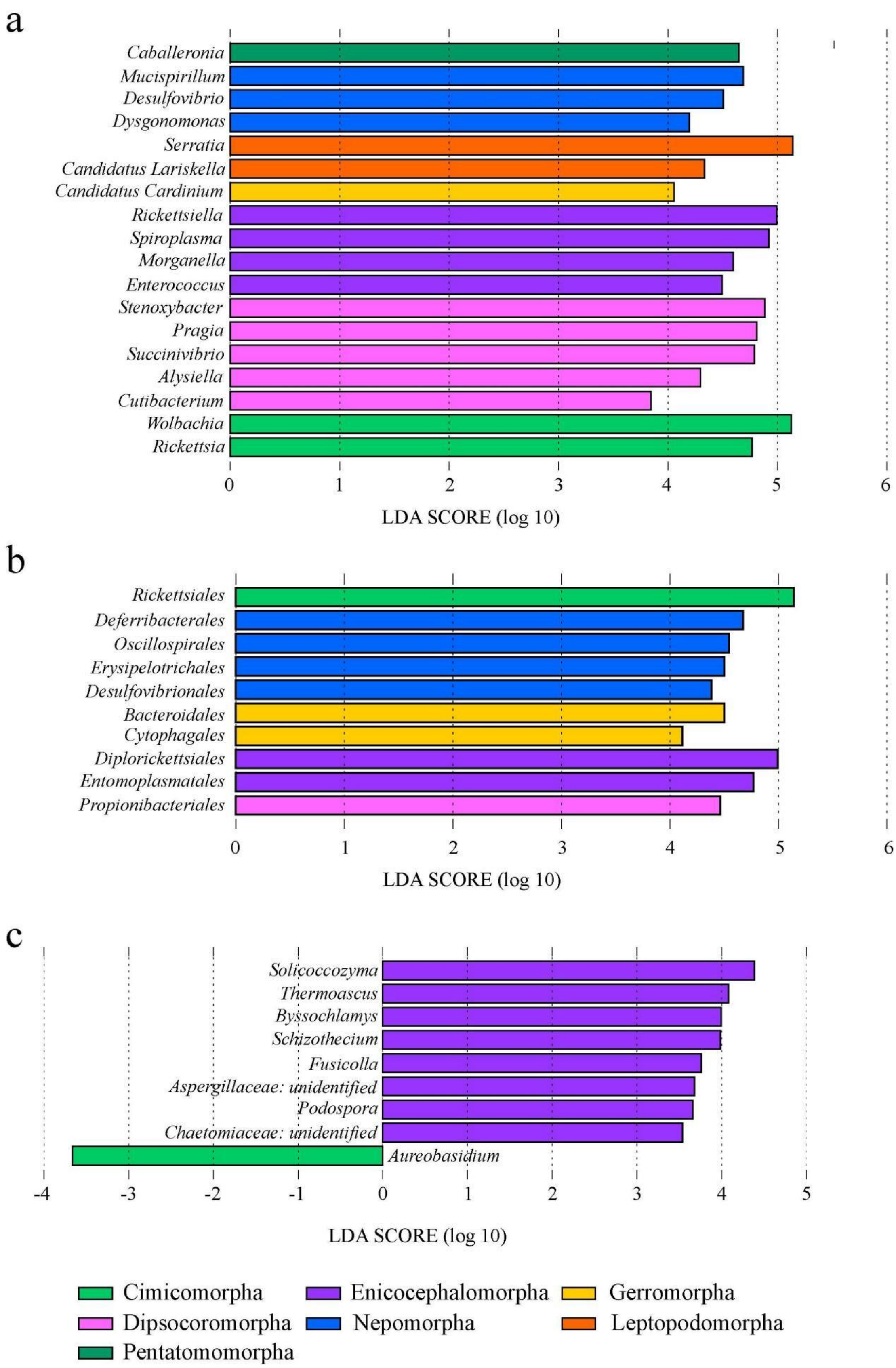

Fig. S10. Significantly different microbial communities in different infraorders.

LEfSe analysis shows the significant differences of bacterial genera (a), bacterial

orders (b), and fungal genera (c) in infraorders. No significantly different fungal order was found. For the unidentified genera, their lowest level classifications are given.

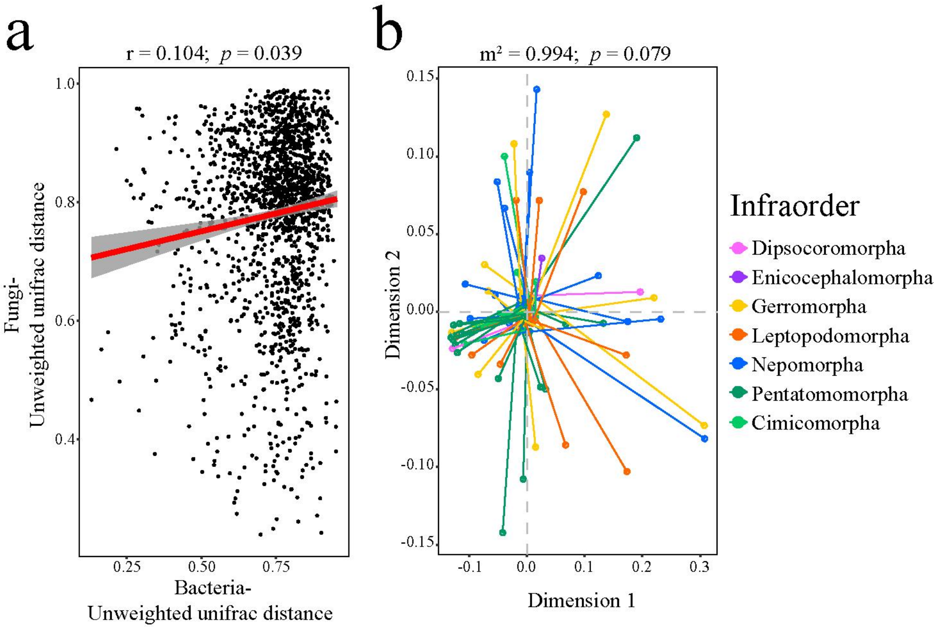

Fig. S11. The Mantel test and Procrustes test between bacterial and fungal communities. The unweighted UniFrac distances of symbiotic bacterial and fungal communities were used to test the relationships between the two kingdoms. (a) Mantel test. (b) Procrustes test.

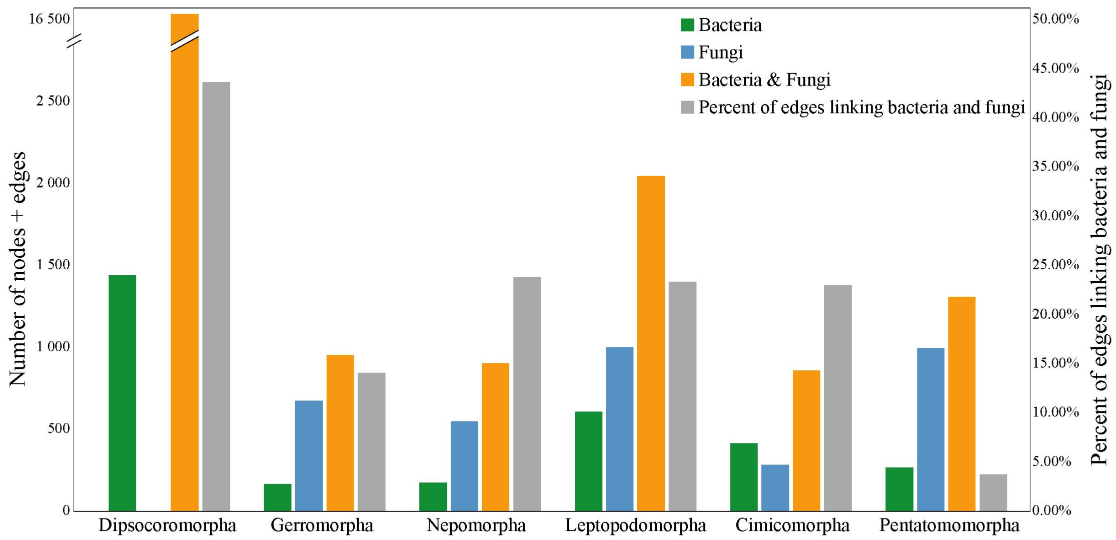

Fig. S12. The number of nodes + edges and the percent of edges linking bacteria and fungi in the networks.

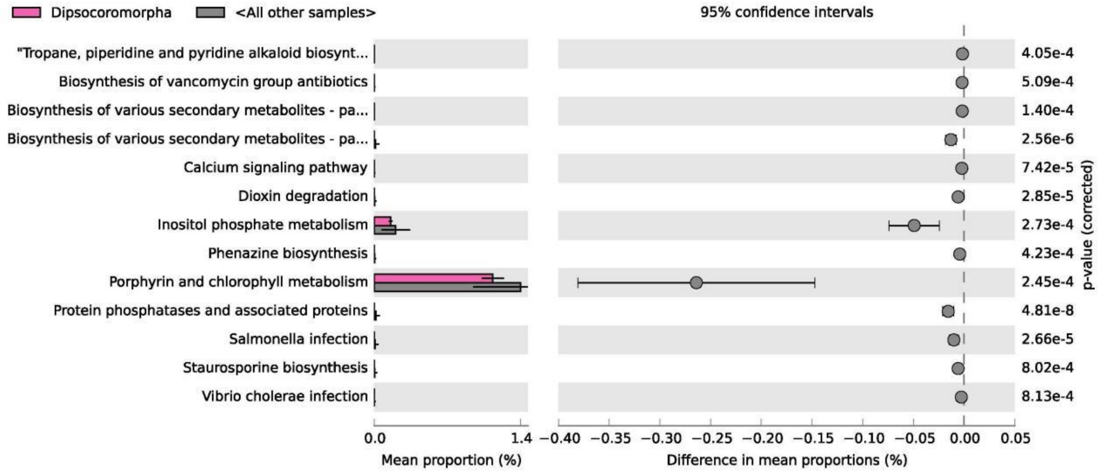

Fig. S13. Functionally predicted pathways differing in proportions between Dipsocoromorpha and all other samples. Only the pathways with p value < 0.001 (Welch's t-test) are shown.

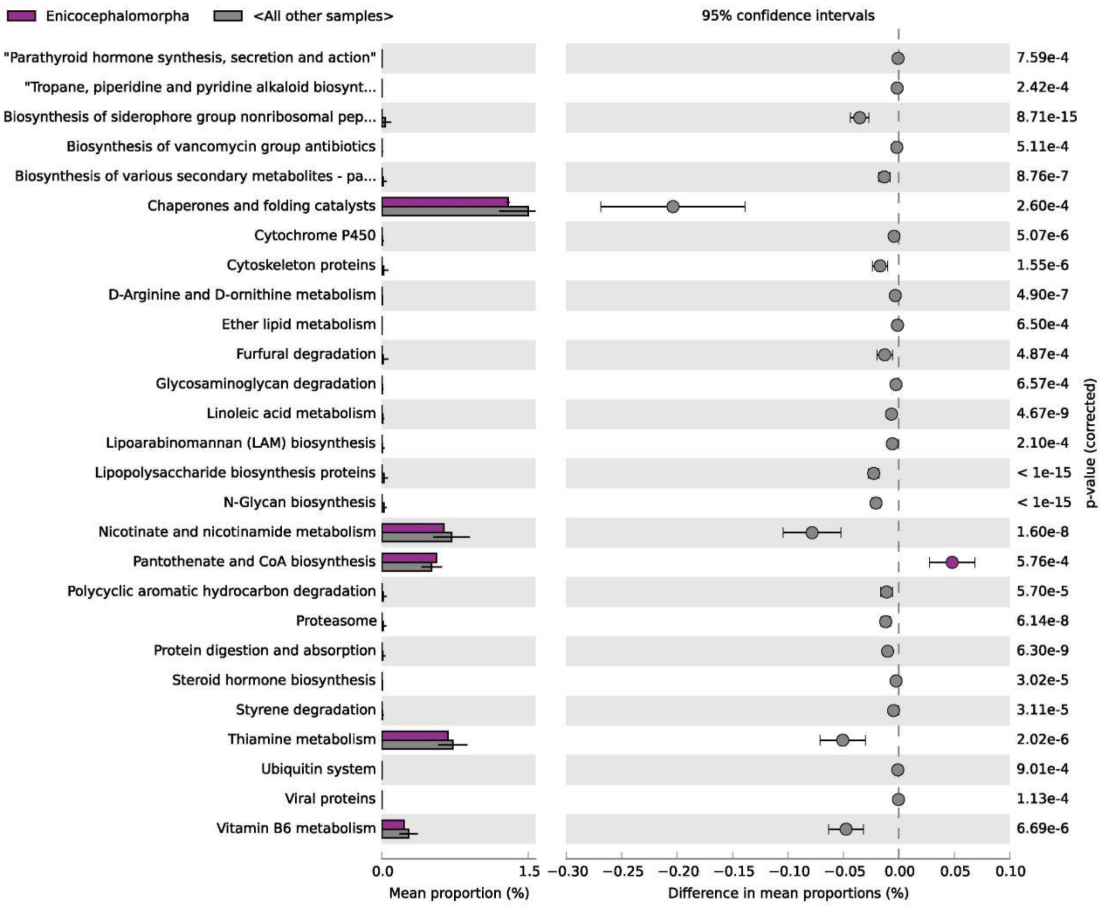

Fig. S14. Functionally predicted pathways differing in proportions between Enicocephalomorpha and all other samples. Only the pathways with *p* value < 0.001 (Welch's t-test) are shown.

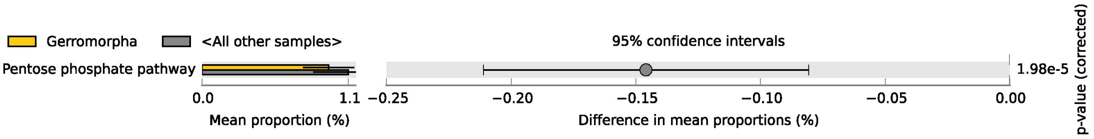

Fig. S15. Functionally predicted pathways differing in proportions between Gerromorpha and all other samples. Only the pathways with *p* value < 0.001 (Welch's t-test) are shown.

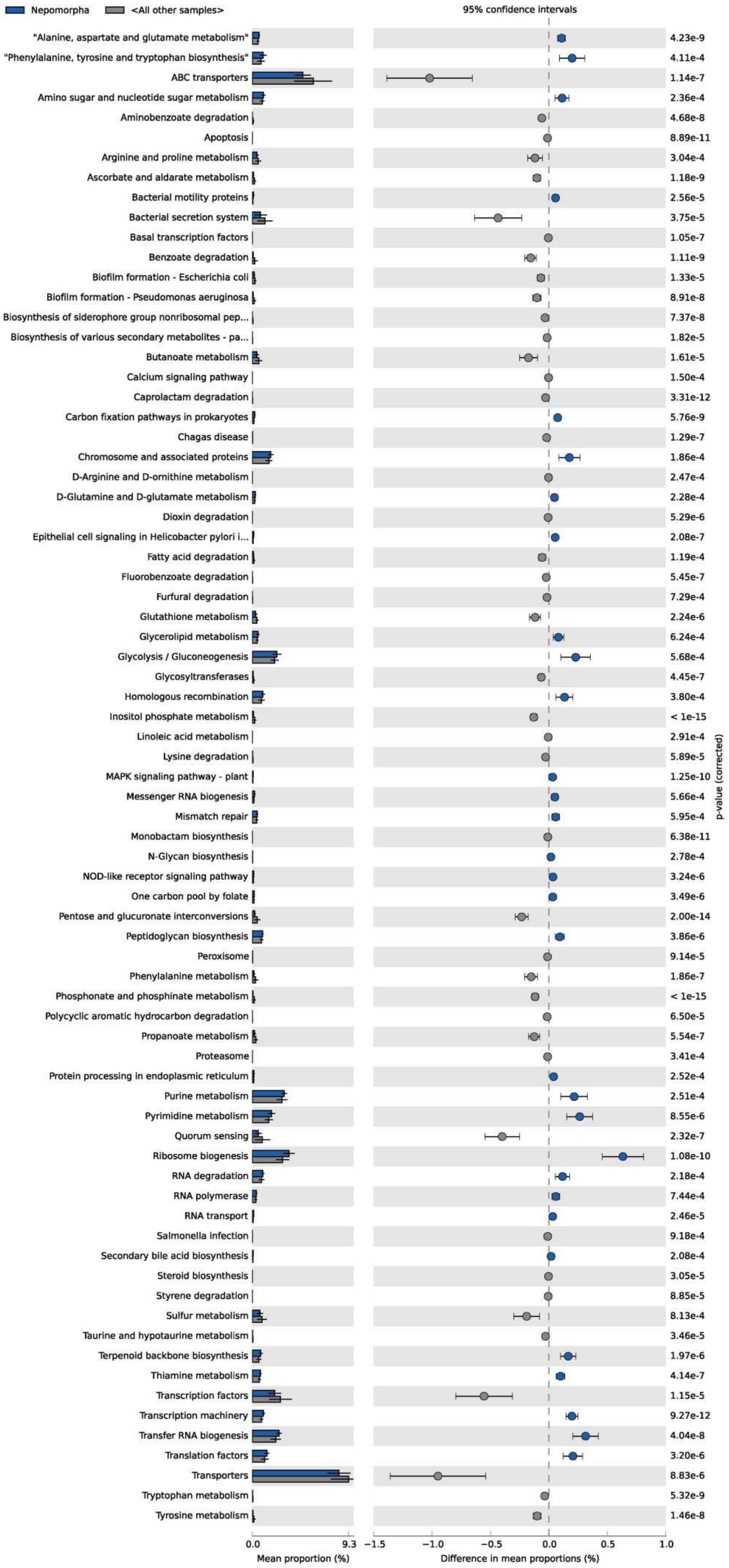
Nepomorpha
<All other samples>
95% confidence intervals
"Alanine, aspartate and glutamate metabolism" 4.23e-9
"Phenylalanine, tyrosine and tryptophan biosynthesis" 4.11e-4
ABC transporters 1.14e-7
Amino sugar and nucleotide sugar metabolism 2.36e-4
Aminobenzoate degradation 4.68e-8
Apoptosis 8.89e-11
Arginine and proline metabolism 3.04e-4
Ascorbate and aldarate metabolism 1.18e-9
Bacterial motility proteins 2.56e-5
Bacterial secretion system 3.75e-5
Basal transcription factors 1.05e-7
Benzoate degradation 1.11e-9
Biofilm formation - Escherichia coli 1.33e-5
Biofilm formation - Pseudomonas aeruginosa 8.91e-8
Biosynthesis of siderophore group nonribosomal pep... 7.37e-8
Biosynthesis of various secondary metabolites - pa... 1.82e-5
Butanoate metabolism 1.61e-5
Calcium signaling pathway 1.50e-4
Caprolactam degradation 3.31e-12
Carbon fixation pathways in prokaryotes 5.76e-9
Chagas disease 1.29e-7
Chromosome and associated proteins 1.86e-4
D-Arginine and D-ornithine metabolism 2.47e-4
D-Glutamine and D-glutamate metabolism 2.28e-4
Dioxin degradation 5.29e-6
Epithelial cell signaling in Helicobacter pylori i... 2.08e-7
Fatty acid degradation 1.19e-4
Fluorobenzoate degradation 5.45e-7
Furfural degradation 7.29e-4
Glutathione metabolism 2.24e-6
Glycerolipid metabolism 6.24e-4
Glycolysis / Gluconeogenesis 5.68e-4
Glycosyltransferases 4.45e-7
Homologous recombination 3.80e-4
Inositol phosphate metabolism < 1e-15
Linoleic acid metabolism 2.91e-4
Lysine degradation 5.89e-5
MAPK signaling pathway - plant 1.25e-10
Messenger RNA biogenesis 5.66e-4
Mismatch repair 5.95e-4
Monobactam biosynthesis 6.38e-11
N-Glycan biosynthesis 2.78e-4
NOD-like receptor signaling pathway 3.24e-6
One carbon pool by folate 3.49e-6
Pentose and glucuronate interconversions 2.00e-14
Peptidoglycan biosynthesis 3.86e-6
Peroxisome 9.14e-5
Phenylalanine metabolism 1.86e-7
Phosphonate and phosphinate metabolism < 1e-15
Polycyclic aromatic hydrocarbon degradation 6.50e-5
Propanoate metabolism 5.54e-7
Proteasome 3.41e-4
Protein processing in endoplasmic reticulum 2.52e-4
Purine metabolism 2.51e-4
Pyrimidine metabolism 8.55e-6
Quorum sensing 2.32e-7
Ribosome biogenesis 1.08e-10
RNA degradation 2.18e-4
RNA polymerase 7.44e-4
RNA transport 2.46e-5
Salmonella infection 9.18e-4
Secondary bile acid biosynthesis 2.08e-4
Steroid biosynthesis 3.05e-5
Styrene degradation 8.85e-5
Sulfur metabolism 8.13e-4
Taurine and hypotaurine metabolism 3.46e-5
Terpenoid backbone biosynthesis 1.97e-6
Thiamine metabolism 4.14e-7
Transcription factors 1.15e-5
Transcription machinery 9.27e-12
Transfer RNA biogenesis 4.04e-8
Translation factors 3.20e-6
Transporters 8.83e-6
Tryptophan metabolism 5.32e-9
Tyrosine metabolism 1.46e-8
p-value (corrected)
0.0
9.3
Mean proportion (%)
−1.5
−1.0
−0.5
0.0
0.5
1.0
Difference in mean proportions (%)

Fig. S16. Functionally predicted pathways differing in proportions between Nepomorpha and all other samples. Only the pathways with p value < 0.001 (Welch's t-test) are shown.

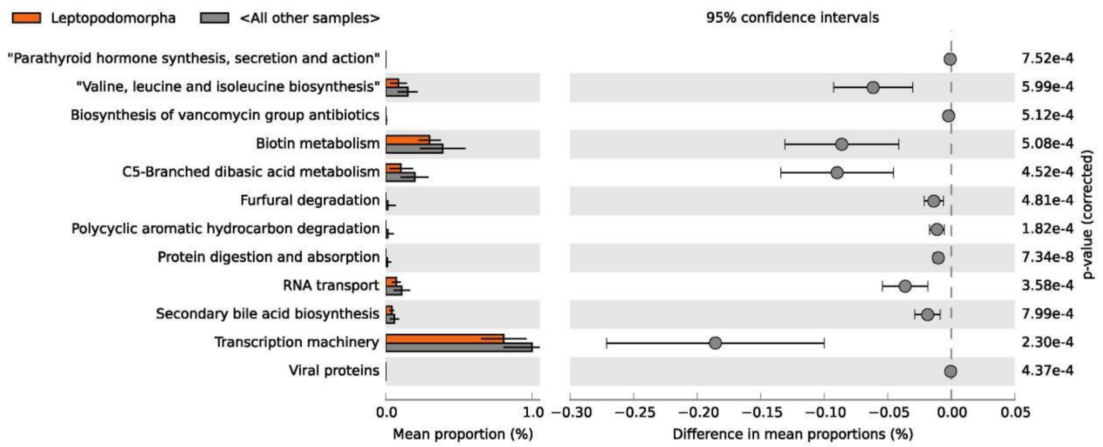

Fig. S17. Functionally predicted pathways differing in proportions between Leptopodomorpha and all other samples. Only the pathways with p value < 0.001 (Welch's t-test) are shown.

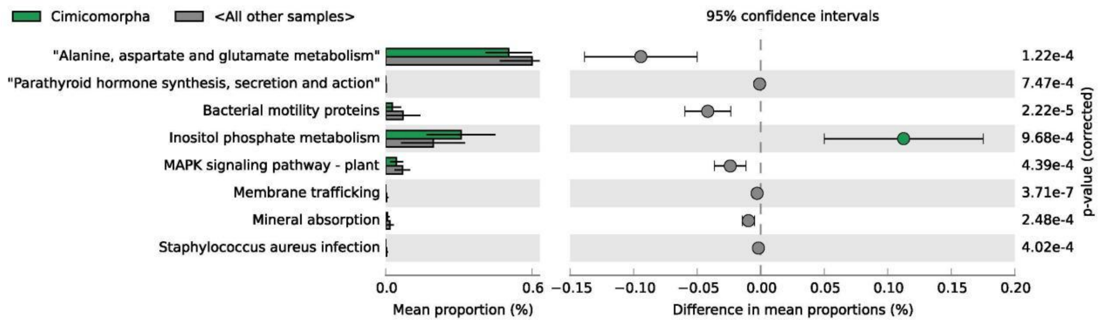

Fig. S18. Functionally predicted pathways differing in proportions between Cimicomorpha and all other samples. Only the pathways with p value < 0.001 (Welch's t-test) are shown.

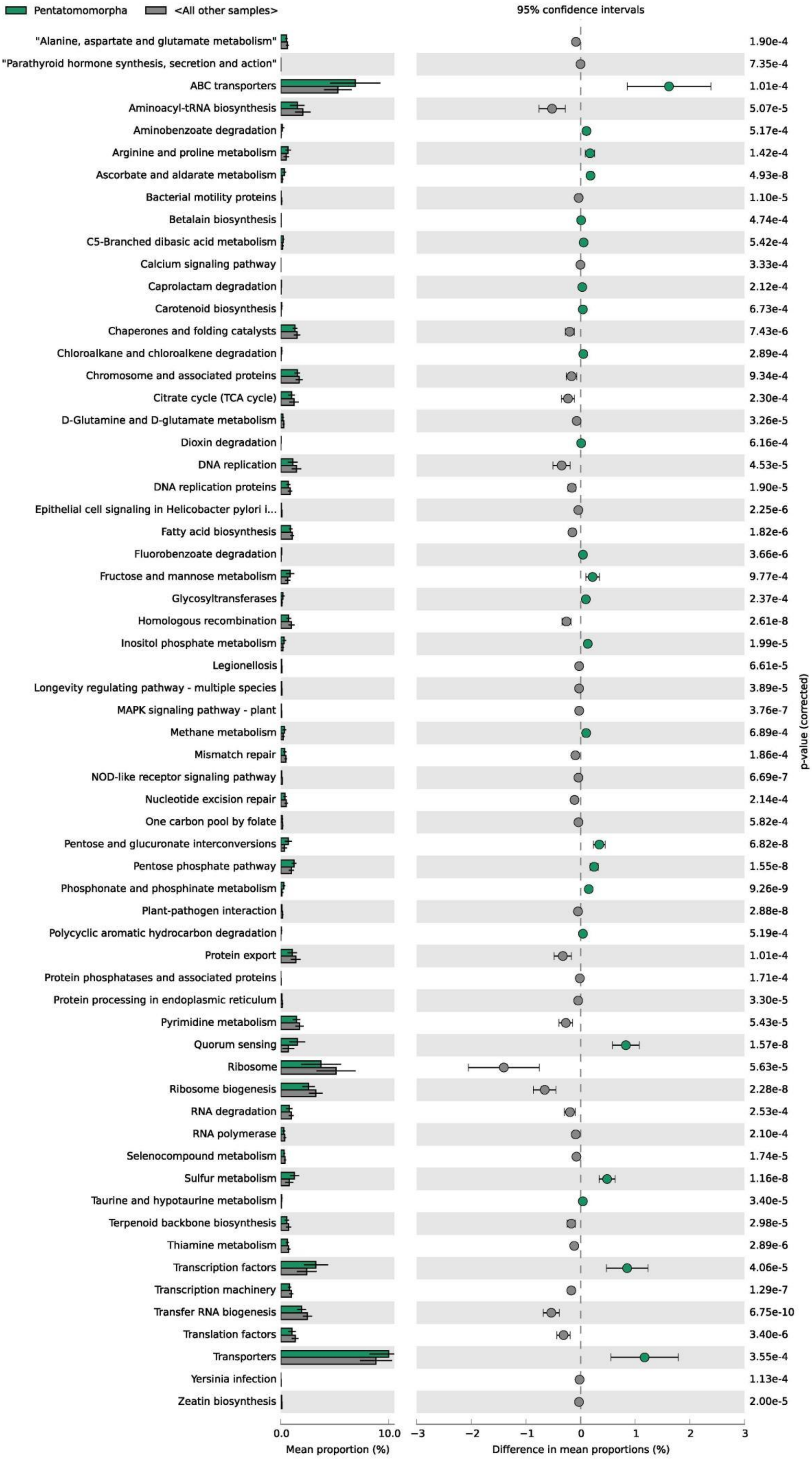
Pentatomomorpha
<All other samples>
95% confidence intervals
"Alanine, aspartate and glutamate metabolism" 1.90e-4
"Parathyroid hormone synthesis, secretion and action" 7.35e-4
ABC transporters 1.01e-4
Aminoacyl-tRNA biosynthesis 5.07e-5
Aminobenzoate degradation 5.17e-4
Arginine and proline metabolism 1.42e-4
Ascorbate and aldarate metabolism 4.93e-8
Bacterial motility proteins 1.10e-5
Betalain biosynthesis 4.74e-4
C5-Branched dibasic acid metabolism 5.42e-4
Calcium signaling pathway 3.33e-4
Caprolactam degradation 2.12e-4
Carotenoid biosynthesis 6.73e-4
Chaperones and folding catalysts 7.43e-6
Chloroalkane and chloroalkene degradation 2.89e-4
Chromosome and associated proteins 9.34e-4
Citrate cycle (TCA cycle) 2.30e-4
D-Glutamine and D-glutamate metabolism 3.26e-5
Dioxin degradation 6.16e-4
DNA replication 4.53e-5
DNA replication proteins 1.90e-5
Epithelial cell signaling in Helicobacter pylori i... 2.25e-6
Fatty acid biosynthesis 1.82e-6
Fluorobenzoate degradation 3.66e-6
Fructose and mannose metabolism 9.77e-4
Glycosyltransferases 2.37e-4
Homologous recombination 2.61e-8
Inositol phosphate metabolism 1.99e-5
Legionellosis 6.61e-5
Longevity regulating pathway - multiple species 3.89e-5
MAPK signaling pathway - plant 3.76e-7
Methane metabolism 6.89e-4
Mismatch repair 1.86e-4
NOD-like receptor signaling pathway 6.69e-7
Nucleotide excision repair 2.14e-4
One carbon pool by folate 5.82e-4
Pentose and glucuronate interconversions 6.82e-8
Pentose phosphate pathway 1.55e-8
Phosphonate and phosphinate metabolism 9.26e-9
Plant-pathogen interaction 2.88e-8
Polycyclic aromatic hydrocarbon degradation 5.19e-4
Protein export 1.01e-4
Protein phosphatases and associated proteins 1.71e-4
Protein processing in endoplasmic reticulum 3.30e-5
Pyrimidine metabolism 5.43e-5
Quorum sensing 1.57e-8
Ribosome 5.63e-5
Ribosome biogenesis 2.28e-8
RNA degradation 2.53e-4
RNA polymerase 2.10e-4
Selenocompound metabolism 1.74e-5
Sulfur metabolism 1.16e-8
Taurine and hypotaurine metabolism 3.40e-5
Terpenoid backbone biosynthesis 2.98e-5
Thiamine metabolism 2.89e-6
Transcription factors 4.06e-5
Transcription machinery 1.29e-7
Transfer RNA biogenesis 6.75e-10
Translation factors 3.40e-6
Transporters 3.55e-4
Yersinia infection 1.13e-4
Zeatin biosynthesis 2.00e-5
p-value (corrected)
0.0
10.0
Mean proportion (%)
−3
−2
−1
0
1
2
3
Difference in mean proportions (%)

Figure S19. Functionally predicted pathways differing in proportions between Pentatomomorpha and all other samples. Only the pathways with p value < 0.001 (Welch's t-test) are shown

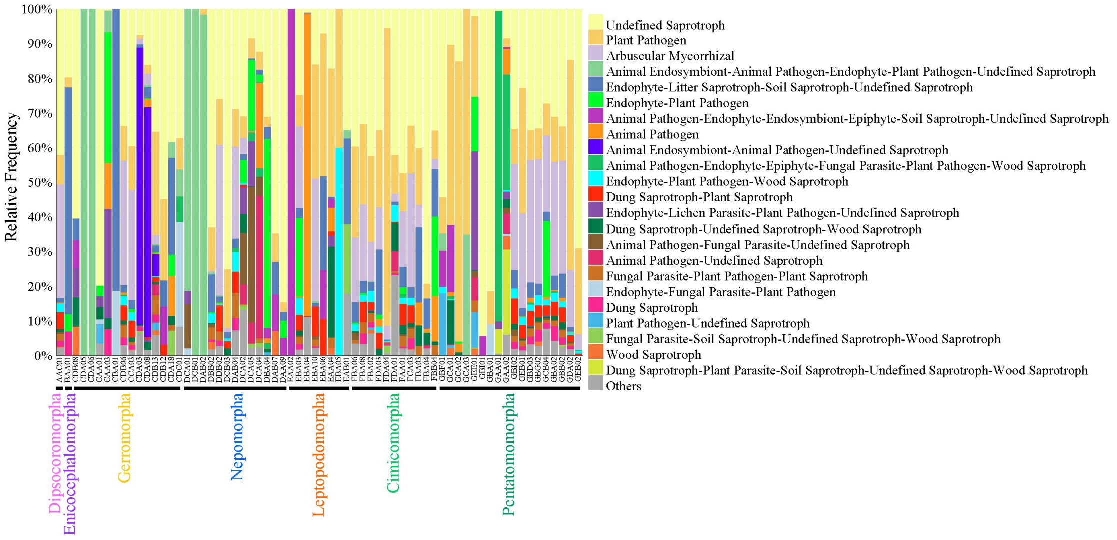

Fig. S20. Composition of functional guilds for fungal communities in true bugs. The relative abundance plots of functional guilds are shown. The infraorders or superfamilies are shown at the bottom.

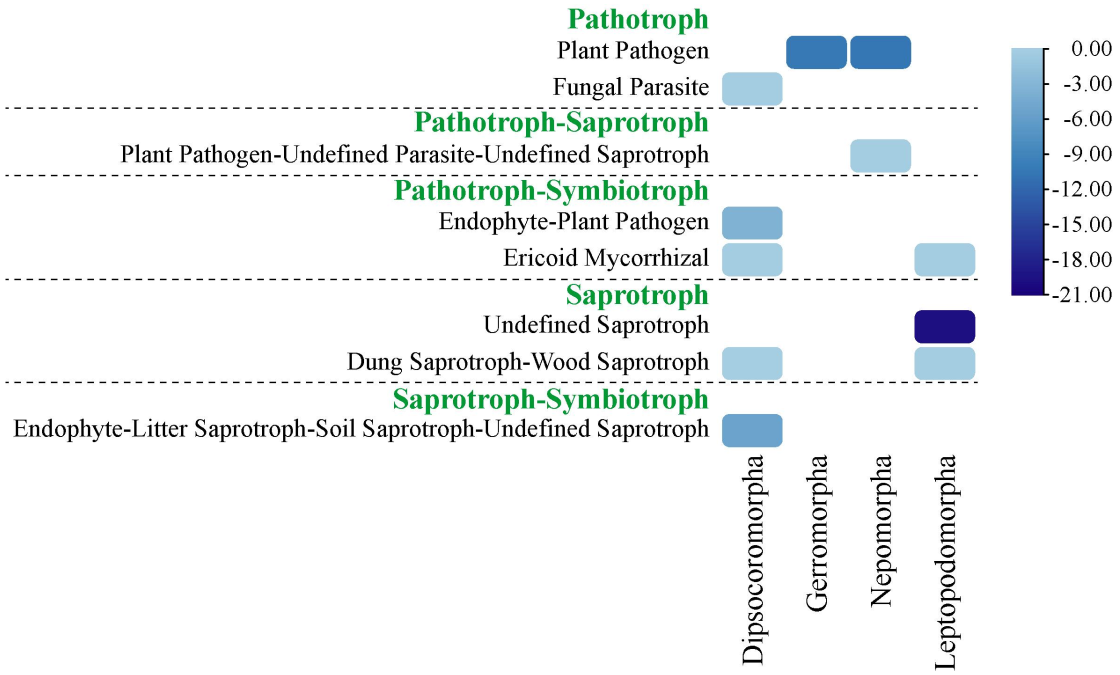

Fig. S21. Heatmap of functionally predicted guilds of symbiotic fungal communities. For each infraorder, the proportions of guilds were compared with those of all other samples. The color represents the difference in mean proportions between a specific infraorder and the remaining samples. Only the guilds with a *p* value < 0.01 (Welch's t test) are shown in the heatmap.

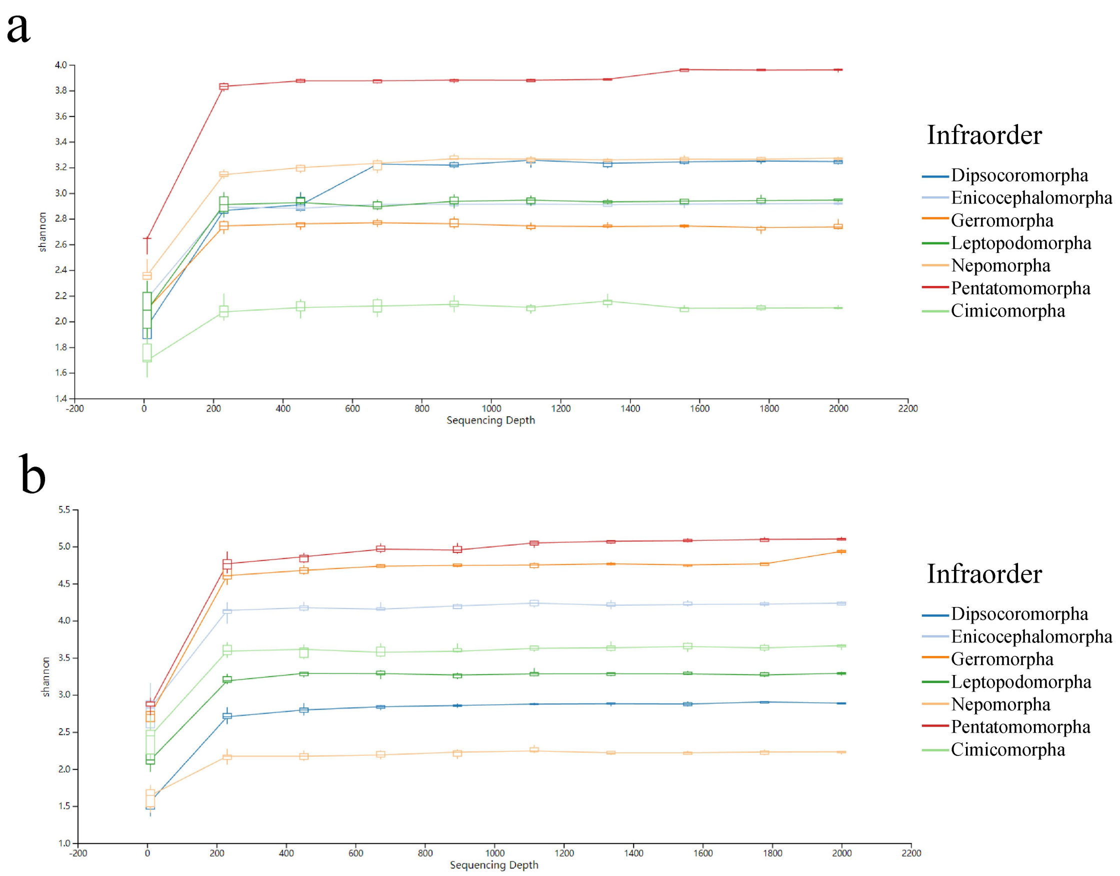

Fig. S22. Rarefaction curves of (a) bacteria and (b) fungi in different infraorders.

Supplementary Tables

Table S1. The collecting and grouping information for all samples.

#SampleID	Infraorder	Superfamily	Family	Species	Area	Temperature zone	Altitude	Collecting date	Province	East longitude	Northern latitude	Collecting Altitude	Fungal sample	Bacterial sample
AAA01-19091801	Dipsocoromorpha		Dipsocoridae	*Cryptostemma* sp.	southeast	marginal tropical zone	low altitude	2019-VII-20-21	Yunnan	101.2092	21.9662	580m	√	√
AAA02-21101908	Dipsocoromorpha		Dipsocoridae	*Cryptostemma* sp.	southeast	marginal tropical zone	low altitude	2021-V-14	Yunnan	101.2768	21.9227	550		√
AAA03-21101909	Dipsocoromorpha		Dipsocoridae	*Cryptostemma* sp.	southeast	south subtropical zone	low altitude	2021-V-10	Yunnan	100.9242	23.2066	845		√
AAB01-19100909	Dipsocoromorpha		Ceratocombidae	*Ceratocombus* sp. 1	southeast	south subtropics zone	low altitude	2019-VIII-29	Yunnan	97.6121	23.9851	857m		√
AAB02-19100908	Dipsocoromorpha		Ceratocombidae	*Ceratocombus* sp. 2	southeast	mid-subtropical zone	middle altitude	2019-VIII-21	Yunnan	97.8575	25.0933	1420m		√
AAB03-21101907	Dipsocoromorpha		Ceratocombidae	*Ceratocombus* sp. 3	southeast	south subtropical zone	sub-high altitude	2021-VIII-24	Yunnan	98.8424	24.6417	2130		√
AAC01-20070612	Dipsocoromorpha		Schizopteridae	*Kokeshia xiei*	southeast	marginal tropical zone	low altitude	2020-V-28	Hainan	109.4133	19.0792	230m	√	√
BAA01-20112101	Enicocephalomorpha		Aenictopecheidae	*Aenictopecheinae* sp.	northwest	plateau temperate zone	sub-high altitude	2020-VIII-12	Tibet	95.5233	29.7017	2620m	√	√
BAB01-20122301	Enicocephalomorpha		Enicocephalidae	*Hoplitocoris lewisi*	northwest	north subtropical zone	middle altitude	2020.10.10~21	Shaanxi	108.7511	33.7769	1291m		√
CAA01-19100912	Gerromorpha	Mesoveloidea	Mesoveliidae	*Mesovelia vittigera*	southeast	south subtropical zone	low altitude	2019-IX-13	Guangxi	106.7081	22.1992	100m	√	√
CAA02-21101807	Gerromorpha	Mesoveloidea	Mesoveliidae	*Mesovelia* sp.	southeast	marginal tropical zone	low altitude	2021-V-13	Yunnan	101.2092	21.9662	580		√
CAA03-19101401	Gerromorpha	Mesoveloidea	Mesoveliidae	*Mesovelia thermalis*	southeast	warm temperate zone	low altitude	2019-IX-28	Tianjin	117.1692	39.1028	0m	√	√
CAA04-20070412	Gerromorpha	Mesoveloidea	Mesoveliidae	*Mesovelia vittigera*	southeast	south subtropical zone	low altitude	2020-V-16	Yunnan	102.1942	22.8533	865m		√
CAA05-20122303	Gerromorpha	Mesoveloidea	Mesoveliidae	*Mesovelia vittigera*	southeast	south subtropical zone	low altitude	2020-XI-28	Guangdong	113.3205	23.0783	9m		√
CBA01-19101402	Gerromorpha	Hebroidea	Hebridae	*Timasius* sp. 1	southeast	mid-subtropical zone	middle altitude	2019-IX-16	Guangxi	109.1606	25.4217	1303m	√	√
CBA02-21101407	Gerromorpha	Hebroidea	Hebridae	*Timasius* sp. 2	southeast	mid-subtropical zone	middle altitude	2021-VIII-21	Yunnan	98.6003	28.0159	1825		√
CBA3-20112114	Gerromorpha	Hebroidea	Hebridae	*Hebrus* sp. 1	southeast	mid-subtropical zone	middle altitude	2020-VIII-23	Yunnan	98.6000	28.0158	1781m		√
CBA04-21101408	Gerromorpha	Hebroidea	Hebridae	*Hebrus* sp. 2	southeast	south subtropical zone	low altitude	2021-V-10	Yunnan	100.9242	23.2066	845		√
CBA05-21101903	Gerromorpha	Hebroidea	Hebridae	*Hebrus* sp. 3	southeast	marginal tropical zone	low altitude	2021-V-13	Yunnan	101.2092	21.9662	580		√
CBA06-20070512	Gerromorpha	Hebroidea	Hebridae	*Hyrcanus* sp.	southeast	marginal tropical zone	low altitude	2020-V-2	Yunnan	101.6311	22.6492	860m		√
CCA01-19082909	Gerromorpha	Hydrometroidea	Hydrometridae	*Hydrometra* sp.	southeast	marginal tropical zone	low altitude	2019-VII-20	Yunnan	101.2523	21.9350	569m		√
CCA02-19090407	Gerromorpha	Hydrometroidea	Hydrometridae	*Hydrometra* sp.	southeast	marginal tropical zone	low altitude	2019-VIII-8	Yunnan	98.9501	23.5120	440m		√
CCA03-20111517	Gerromorpha	Hydrometroidea	Hydrometridae	*Hydrometra greeni*	southeast	south subtropical zone	low altitude	2020-IX-3	Yunnan	97.5786	24.4478	380m	√	√
CCA04-21101806	Gerromorpha	Hydrometroidea	Hydrometridae	*Hydrometra greeni*	southeast	marginal tropical zone	low altitude	2021-V-19	Yunnan	101.4516	21.4068	607		√
CDA01-19082911	Gerromorpha	Gerroidea	Veliidae	*Microvelia* sp. 4	southeast	south subtropical zone	low altitude	2019-VII-18	Yunnan	101.0205	22.5837	895m		√

CDA02-19082912	Gerromorpha	Gerroidea	Veliidae	*Perittopus crinalis*	southeast	marginal tropical zone	middle altitude	2019-VII-26	Yunnan	100.5014	21.6257	1188m		√
CDA03-20112115	Gerromorpha	Gerroidea	Veliidae	*Perittopus asiaticus*	southeast	south subtropical zone	low altitude	2020-IX-3	Yunnan	97.5786	24.4478	380m	√	√
CDA04-19082908	Gerromorpha	Gerroidea	Veliidae	*Microvelia (Picaultia) douglasi*	southeast	mid-subtropical zone	middle altitude	2019-VIII-15	Yunnan	98.7944	25.2949	1560m		√
CDA05-19092310	Gerromorpha	Gerroidea	Veliidae	*Microvelia* sp.	southeast	marginal tropical zone	middle altitude	2019-VIII-5	Yunnan	99.0860	23.2518	1163m	√	√
CDA06-19092311	Gerromorpha	Gerroidea	Veliidae	*Microvelia (Picaultia)* sp.	southeast	marginal tropical zone	low altitude	2019-VIII-5	Yunnan	99.0739	23.2739	566m	√	√
CDA07-20112108	Gerromorpha	Gerroidea	Veliidae	*Microvelia (Picaultia)* sp.	southeast	mid-subtropical zone	low altitude	2020-X-15	Sichuan	104.7180	31.9086	791m		√
CDA08-20112116	Gerromorpha	Gerroidea	Veliidae	*Pseudovelia* sp.	southeast	south subtropical zone	low altitude	2020-IX-3	Yunnan	97.5786	24.4478	380m	√	√
CDA09-20122304	Gerromorpha	Gerroidea	Veliidae	*Microvelia kyushuensis*	southeast	south subtropical zone	low altitude	2020-XI-28	Guangdong	113.3205	23.0783	9m		√
CDA10-21101409	Gerromorpha	Gerroidea	Veliidae	*Microvelia* sp.	southeast	marginal tropical zone	low altitude	2021-V-14	Yunnan	101.2768	21.9227	550		√
CDA11-21101410	Gerromorpha	Gerroidea	Veliidae	*Microvelia* sp. 2	southeast	mid-subtropical zone	middle altitude	2021-IV-25	Sichuan	102.3753	27.2687	1669		√
CDA12-20070510	Gerromorpha	Gerroidea	Veliidae	*Baptista* sp. 1	southeast	marginal tropical zone	low altitude	2020-V-2	Yunnan	101.6311	22.6492	860m		√
CDA13-20070509	Gerromorpha	Gerroidea	Veliidae	*Microvelia* sp. 3	southeast	marginal tropical zone	low altitude	2020-V-26	Hainan	109.6706	18.9056	720m		√
CDA14-21101906	Gerromorpha	Gerroidea	Veliidae	*Pseudobaptista* sp.	southeast	mid-subtropical zone	low altitude	2021-VII-21	Guangdong	112.3389	25.0897	315		√
CDA15-20070511	Gerromorpha	Gerroidea	Veliidae	*Rhagovelia* sp.1	southeast	marginal tropical zone	low altitude	2020-V-2	Yunnan	101.6311	22.6492	860m		√
CDA16-20070411	Gerromorpha	Gerroidea	Veliidae	*Rhagovelia* sp.2	southeast	marginal tropical zone	low altitude	2020-V-26	Hainan	109.6706	18.9056	720m		√
CDA17-21101904	Gerromorpha	Gerroidea	Veliidae	*Rhagovelia* sp.3	southeast	marginal tropical zone	low altitude	2021-V-13	Yunnan	101.2092	21.9662	580		√
CDA18-Halovelia	Gerromorpha	Gerroidea	Veliidae	*Halovelia* sp.	southeast	tropical zone	low altitude	2019-V-5~6	Hainan	111.6061	16.4473	12.35m	√	√
CDB01-21101911	Gerromorpha	Gerroidea	Gerridae	Gerridae sp.	southeast	marginal tropical zone	low altitude	2021-V-19	Yunnan	101.4516	21.4068	607		√
CDB02-21101811	Gerromorpha	Gerroidea	Gerridae	*Amemboa* sp.1	southeast	marginal tropical zone	low altitude	2021-V-17	Yunnan	101.5496	21.4915	674		√
CDB03-21101812	Gerromorpha	Gerroidea	Gerridae	*Amemboa* sp.2	southeast	marginal tropical zone	low altitude	2021-V-20	Yunnan	101.2994	21.5302	550		√
CDB04-19082910	Gerromorpha	Gerroidea	Gerridae	*Ptilomera tigrina*	southeast	south subtropical zone	low altitude	2019-VII-18	Yunnan	101.0205	22.5837	895m		√
CDB05-20070407	Gerromorpha	Gerroidea	Gerridae	*Ptilomera tigrina*	southeast	marginal tropical zone	low altitude	2020-V-5	Yunnan	102.2872	22.5833	320m		√
CDB06-20111307	Gerromorpha	Gerroidea	Gerridae	*Potamometra* sp.1	northwest	north subtropical zone	middle altitude	2020-VIII-15	Shaanxi	108.8064	33.7688	1334m	√	√
CDB07-20111308	Gerromorpha	Gerroidea	Gerridae	*Potamometra* sp.2	northwest	warm temperate zone	low altitude	2020-VIII-21	Shanxi	112.4408	35.2736	658m		√
CDB08-19082907	Gerromorpha	Gerroidea	Gerridae	*Eotrechus siamensis*	southeast	marginal tropical zone	middle altitude	2019-VII-26	Yunnan	100.5014	21.6257	1188m	√	√
CDB09-20070408	Gerromorpha	Gerroidea	Gerridae	*Limnometra* sp.	southeast	marginal tropical zone	low altitude	2020-V-2	Yunnan	101.6311	22.6492	860m		√
CDB10-20112117	Gerromorpha	Gerroidea	Gerridae	*Aquarius paludum*	northwest	warm temperate zone	middle altitude	2020.8.24	Shanxi	112.8376	35.5632	1001m		√
CDB11-20112401	Gerromorpha	Gerroidea	Gerridae	*Aquarius* sp.	northwest	warm temperate zone	low altitude	2020-VIII-21	Shanxi	112.4408	35.2736	658m		√
CDB12-21101308	Gerromorpha	Gerroidea	Gerridae	*Aquarius paludum*	southeast	mid-subtropical zone	middle altitude	2021-V-3	Sichuan	101.4635	26.8075	1333	√	√
CDB13-20112119	Gerromorpha	Gerroidea	Gerridae	*Gerris (Macrogerris) tigrinus*	northwest	warm temperate zone	sub-high altitude	2020-VII-27	Ningxia	106.3508	35.3173	2191m	√	√
CDB14-20112118	Gerromorpha	Gerroidea	Gerridae	*Gerris (Macrogerris) gracilicornis*	northwest	plateau temperate zone	sub-high altitude	2020-VIII-12	Tibet	95.5233	29.7017	2620m		√

CDB15-21101305	Gerromorpha	Gerroidea	Gerridae	*Gerris latiabdominis*	southeast	mid-subtropical zone	middle altitude	2021-IV-25	Sichuan	102.3753	27.2687	1669		√
CDB16-20122302	Gerromorpha	Gerroidea	Gerridae	*Limnogonus nitidus*	southeast	south subtropical zone	low altitude	2020-XI-28	Guangdong	113.3205	23.0783	9m		√
CDB17-21101405	Gerromorpha	Gerroidea	Gerridae	*Limnogonus fossarum fossarum*	southeast	marginal tropical zone	low altitude	2021-V-19	Yunnan	101.4516	21.4068	607		√
CDB18-21101809	Gerromorpha	Gerroidea	Gerridae	*Limnogonus fossarum fossarum*	southeast	mid-subtropical zone	middle altitude	2021-V-1	Sichuan	101.8084	26.7256	1247		√
CDB19-21101912	Gerromorpha	Gerroidea	Gerridae	*Limnogonus nitidus*	southeast	marginal tropical zone	low altitude	2021-V-19	Yunnan	101.4516	21.4068	607		√
CDB20-21101406	Gerromorpha	Gerroidea	Gerridae	*Limnometra matsudai*	southeast	marginal tropical zone	low altitude	2021-V-14	Yunnan	101.2768	21.9227	550		√
CDB21-21101307	Gerromorpha	Gerroidea	Gerridae	*Metrocoris* sp.	southeast	mid-subtropical zone	middle altitude	2021-IV-29	Sichuan	101.9118	27.0993	1410		√
CDB22-21033102	Gerromorpha	Gerroidea	Gerridae	*Gigantometra gigas*	southeast	south subtropical zone	low altitude	2021-III-20	Guangdong	111.9067	23.4656	275m		√
CDC01-Hermatonates	Gerromorpha	Gerroidea	Hermatobatidae	*Hermatobates lingyangjiaoensis*	southeast	tropical zone	low altitude	2019-XI-12	Hainan	111.6061	16.4473	12.35m	√	√
DAA01-19112507	Nepomorpha	Corixoidea	Micronectidae	*Micronecta* sp. 1	southeast	south subtropics zone	low altitude	2019-VII-16-17	Guangxi	108.2694	22.1333	2m		√
DAA02-19112508	Nepomorpha	Corixoidea	Micronectidae	*Micronecta* sp. 2	southeast	marginal tropical zone	low altitude	2019-VII-30	Yunnan	99.6822	22.1360	940m		√
DAA03-19112509	Nepomorpha	Corixoidea	Micronectidae	*Micronecta* sp. 3	southeast	mid-subtropical zone	low altitude	2019-IX-18	Guangdong	114.2602	24.7154	397m		√
DAA04-19112510	Nepomorpha	Corixoidea	Micronectidae	*Micronecta* sp. 4	southeast	south subtropical zone	low altitude	2019-IX-13	Guangxi	106.7081	22.1992	100m		√
DAA05-21012805	Nepomorpha	Corixoidea	Micronectidae	*Micronecta* sp. 5	southeast	south subtropical zone	low altitude	2021-I-26	Guangdong	113.3205	23.0783	9m		√
DAA06-21012802	Nepomorpha	Corixoidea	Micronectidae	*Micronecta* sp. 6	southeast	south subtropical zone	low altitude	2021-I-26	Guangdong	113.3205	23.0783	9m		√
DAA07-21101808	Nepomorpha	Corixoidea	Micronectidae	*Micronecta* sp. 7	southeast	mid-subtropical zone	middle altitude	2021-V-4	Sichuan	101.4468	26.8271	1228		√
DAA08-21101403	Nepomorpha	Corixoidea	Micronectidae	*Micronecta guttatostriata*	southeast	marginal tropical zone	low altitude	2021-V-17	Yunnan	101.5496	21.4915	674		√
DAA09-21101404	Nepomorpha	Corixoidea	Micronectidae	*Micronecta* sp. 9	southeast	mid-subtropical zone	middle altitude	2021-IV-27	Sichuan	102.2416	27.0561	1414	√	√
DAB01-19090412	Nepomorpha	Corixoidea	Corixidae	*Sigara* sp.3	southeast	marginal tropical zone	low altitude	2019-VII-25	Yunnan	100.2813	21.5819	857m		√
DAB02-19092506	Nepomorpha	Corixoidea	Corixidae	*Cenocorixa* sp. 1	southeast	south subtropics zone	middle altitude	2019-VIII-1	Yunnan	100.3373	22.6279	1203m	√	√
DAB03-20112106	Nepomorpha	Corixoidea	Corixidae	*Sigara* sp.3	northwest	temperate zone	low altitude	2020-VIII-6	InnerMongolia	101.0752	41.9647	929m		√
DAB04-20112110	Nepomorpha	Corixoidea	Corixidae	*Cenocorixa* sp. 2	northwest	plateau temperate zone	sub-high altitude	2020-VIII-12	Tibet	95.5233	29.7017	2620m	√	√
DAB05-20112105	Nepomorpha	Corixoidea	Corixidae	*Sigara* sp.1	northwest	temperate zone	low altitude	2020-VIII-6	InnerMongolia	101.0752	41.9647	929m		√
DAB06-20112103	Nepomorpha	Corixoidea	Corixidae	*Sigara* sp.2	northwest	temperate zone	low altitude	2020-VIII-6	InnerMongolia	101.0752	41.9647	929m		√
DAB07-21101306	Nepomorpha	Corixoidea	Corixidae	*Sigara* sp.3	southeast	mid-subtropical zone	middle altitude	2021-IV-28	Sichuan	102.2780	27.0789	1563	√	√
DAB08-20112104	Nepomorpha	Corixoidea	Corixidae	*Callicorixa* sp.	northwest	temperate zone	middle altitude	2020-VII-29	Ningxia	105.9166	38.7488	1937m		√
DAB09-20112102	Nepomorpha	Corixoidea	Corixidae	*Cymatia rogenhoferi*	northwest	temperate zone	low altitude	2020-VIII-6	InnerMongolia	101.0752	41.9647	929m		√
DBA01-19091803	Nepomorpha	Nepoidea	Nepidae	*Ranatra* sp. 1	southeast	marginal tropical zone	low altitude	2019-VII-30	Yunnan	99.6822	22.1360	940m		√
DBA02-20070410	Nepomorpha	Nepoidea	Nepidae	*Ranatra* sp. 2	southeast	marginal tropical zone	low altitude	2020-V-2	Yunnan	101.6311	22.6492	860m		√
DBA03-21101303	Nepomorpha	Nepoidea	Nepidae	*Ranatra sterea*	southeast	marginal tropical zone	low altitude	2021-V-20	Yunnan	101.4516	21.4068	607		√
DBA04-21101304	Nepomorpha	Nepoidea	Nepidae	*Cercotmetus asiaticus*	southeast	marginal tropical zone	low altitude	2021-V-19	Yunnan	101.4516	21.4068	607	√	√

DBA05-19091804	Nepomorpha	Nepoidea	Nepidae	*Laccotrephes japonensis*	southeast	south subtropics zone	middle altitude	2019-VIII-1	Yunnan	100.3373	22.6279	1203m		√
DBA06-19091805	Nepomorpha	Nepoidea	Nepidae	*Laccotrephes japonensis*	southeast	south subtropics zone	middle altitude	2019-VIII-1	Yunnan	100.3373	22.6279	1203m		√
DBA07-19091806	Nepomorpha	Nepoidea	Nepidae	*Laccotrephes japonensis*	southeast	south subtropics zone	middle altitude	2019-VIII-1	Yunnan	100.3373	22.6279	1203m		√
DBB01-19102101	Nepomorpha	Nepoidea	Belostomatidae	*Diplonychus* sp. 1	southeast	south subtropical zone	low altitude	2019-X-15	Guangdong	113.2903	23.0958	4m		√
DBB02-20070409	Nepomorpha	Nepoidea	Belostomatidae	*Diplonychus* sp. 2	southeast	marginal tropical zone	low altitude	2020-V-5	Yunnan	102.2872	22.5833	320m	√	√
DBB03-19112512	Nepomorpha	Nepoidea	Belostomatidae	*Lethocerus deyrollei*	southeast	warm temperate zone	low altitude	2019-XI-8	Anhui	116.0000	33.0000			√
DCA01-19092504	Nepomorpha	Ochteroidea	Gelastocoridae	*Nerthra indica*	southeast	marginal tropical zone	low altitude	2019-VII-25	Yunnan	100.2813	21.5819	857m	√	√
DCA02-20112111	Nepomorpha	Ochteroidea	Gelastocoridae	*Nerthra indica*	northwest	mid-subtropical zone	low altitude	2020-VIII-10	Tibet	95.2563	29.2817	672m	√	√
DCA03-21101301	Nepomorpha	Ochteroidea	Gelastocoridae	*Nerthra indica*	southeast	mid-subtropical zone	low altitude	2021-VII-12	Guizhou	107.9378	25.2919	739	√	√
DCA04-21101302	Nepomorpha	Ochteroidea	Gelastocoridae	*Nerthra indica*	southeast	mid-subtropical zone	low altitude	2021-VII-24	Guangdong	113.0269	24.9206	826	√	√
DCB01-19092505	Nepomorpha	Ochteroidea	Ochteridae	*Ochterus* sp. 1	southeast	south subtropics zone	low altitude	2019-VII-17	Guangxi	108.2694	22.1333	2m	√	√
DCB02-19090410	Nepomorpha	Ochteroidea	Ochteridae	*Ochterus* sp. 2	southeast	marginal tropical zone	low altitude	2019-VII-26	Yunnan	100.2813	21.5819	857m		√
DCB03-20111524	Nepomorpha	Ochteroidea	Ochteridae	*Ochterus* sp. 3	southeast	south subtropical zone	low altitude	2020-IX-3	Yunnan	97.5786	24.4478	380m	√	√
DDA01-19100910	Nepomorpha	Notonectoidea	Notonectidae	*Notonecta* sp. 1	southeast	mid-subtropical zone	middle altitude	2019-VIII-18	Yunnan	98.0244	25.2300	1780m		√
DDA02-20111310	Nepomorpha	Notonectoidea	Notonectidae	*Notonecta glauca*	northwest	temperate zone	middle altitude	2020-VII-30	Ningxia	105.9166	38.7488	1937m		√
DDA03-21101401	Nepomorpha	Notonectoidea	Notonectidae	*Notonecta* sp. 3	southeast	mid-subtropical zone	low altitude	2021-VII-15	Chongqing	106.4206	28.6753	927		√
DDA04-21101801	Nepomorpha	Notonectoidea	Notonectidae	*Notonecta* sp. 2	southeast	mid-subtropical zone	sub-high altitude	2021-IV-23	Sichuan	102.4242	27.5810	2125		√
DDA05-20111309	Nepomorpha	Notonectoidea	Notonectidae	*Notonecta chinensis*	northwest	warm temperate zone	low altitude	2020-VIII-25	Shanxi	112.8898	35.4913	761m		√
DDA06-19090408	Nepomorpha	Notonectoidea	Notonectidae	*Enithares* sp.	southeast	marginal tropical zone	low altitude	2019-VII-23	Yunnan	100.6864	21.6004	744m		√
DDA07-19090411	Nepomorpha	Notonectoidea	Notonectidae	*Anisops* sp. 1	southeast	marginal tropical zone	low altitude	2019-VII-23	Yunnan	100.6864	21.6004	744m		√
DDA08-20111518	Nepomorpha	Notonectoidea	Notonectidae	*Anisops ogasawarensis*	northwest	warm temperate zone	low altitude	2020-VIII-25	Shanxi	112.8898	35.4913	761m		√
DDA09-20111311	Nepomorpha	Notonectoidea	Notonectidae	*Anisops elstoni*	southeast	mid-subtropical zone	low altitude	2020-X-15	Sichuan	104.7180	31.9086	791m		√
DDA10-21101402	Nepomorpha	Notonectoidea	Notonectidae	*Anisops kuroiwae*	southeast	south subtropical zone	low altitude	2021-V-9	Yunnan	100.8695	23.2754	972		√
DDB01-19100911	Nepomorpha	Notonectoidea	Helotrephidae	*Helotrephes* sp. 1	southeast	mid-subtropical zone	middle altitude	2019-IX-16	Guangxi	109.1606	25.4217	1303m		√
DDB02-20070507	Nepomorpha	Notonectoidea	Helotrephidae	*Helotrephes* sp. 2	southeast	marginal tropical zone	middle altitude	2020-V-15	Yunnan	102.2492	22.8283	1010m	√	√
DDB03-21101803	Nepomorpha	Notonectoidea	Helotrephidae	*Helotrephes* sp. 3	southeast	mid-subtropical zone	low altitude	2021-VII-24	Guangdong	112.9908	24.6864	580		√
DDB04-21101804	Nepomorpha	Notonectoidea	Helotrephidae	*Helotrephes tuberculatus*	southeast	mid-subtropical zone	low altitude	2021-VII-24	Guangdong	112.9908	24.6864	580		√
DDC01-19101403	Nepomorpha	Notonectoidea	Pleidae	*Paraplea indistinguenda*	southeast	warm temperate zone	low altitude	2019-IX-28	Tianjin	117.1692	39.1028	0m		√
DDC02-21101805	Nepomorpha	Notonectoidea	Pleidae	*Paraplea liturata*	southeast	marginal tropical zone	low altitude	2021-V-14	Yunnan	101.2768	21.9227	550		√
DEA01-20070508	Nepomorpha	Naucoroidea	Naucoridae	*Gestroiella* sp. 1	southeast	marginal tropical zone	low altitude	2020-V-2	Yunnan	101.6311	22.6492	860m		√
DEA02-21101910	Nepomorpha	Naucoroidea	Naucoridae	*Gestroiella* sp. 2	southeast	marginal tropical zone	low altitude	2021-V-19	Yunnan	101.4516	21.4068	607		√

DEA03-21101701	Nepomorpha	Naucoroidea	Naucoridae	*Thurselinus scutellaris*	southeast	marginal tropical zone	low altitude	2021-V-19	Yunnan	101.4516	21.4068	607		√
DEA04-19092312	Nepomorpha	Naucoroidea	Naucoridae	*Thurselinus scutellaris*	southeast	marginal tropical zone	low altitude	2019-VII-20	Yunnan	101.2523	21.9350	569m	√	√
DEB01-20070611	Nepomorpha	Naucoroidea	Aphelocheiridae	*Aphelocheirus* sp. 1	southeast	marginal tropical zone	low altitude	2020-V-26	Hainan	109.6706	18.9056	720m		√
DEB02-21101802	Nepomorpha	Naucoroidea	Aphelocheiridae	*Aphelocheirus* sp. 2	southeast	mid-subtropical zone	middle altitude	2021-IV-30	Sichuan	101.9118	27.0993	1410		√
DEC01-19121101	Nepomorpha	Naucoroidea	Potamocoridae	*Potamocoris* sp.				2016-XII -15		44.8202	22.0423	923m	√	√
EAA01-19091802	Leptopodomorpha	Leptopodoidea	Leptopodidae	*Valleriola* sp.	southeast	south subtropical zone	low altitude	2019-VII-18	Yunnan	101.0205	22.5837	895m	√	√
EAA02-19092503	Leptopodomorpha	Leptopodoidea	Leptopodidae	*Leptopus* sp.	southeast	marginal tropical zone	low altitude	2019-VII-21	Yunnan	101.2078	21.9665	594m	√	√
EAA03-19090409	Leptopodomorpha	Leptopodoidea	Leptopodidae	*Valleriola javanica*	southeast	marginal tropical zone	low altitude	2019-VII-25	Yunnan	100.2813	21.5819	857m		√
EAA04-21101901	Leptopodomorpha	Leptopodoidea	Leptopodidae	*Leptopus* sp.	southeast	mid-subtropical zone	middle altitude	2021-IV-29	Sichuan	101.9118	27.0993	1410	√	√
EAB01-Omaniidae	Leptopodomorpha	Leptopodoidea	Omaniidae	*Corallocoris xishaensis*	southeast	tropical zone	low altitude	2019-IV-18	Hainan	112.3461	16.8442	5m	√	√
EBA01-19112511	Leptopodomorpha	Saldoidea	Saldidae	*Saldula* sp. 1	southeast	south subtropical zone	low altitude	2019-VIII-27	Yunnan	97.9942	24.4450	955m		√
EBA02-20111312	Leptopodomorpha	Saldoidea	Saldidae	*Saldula* sp. 2	northwest	temperate zone	low altitude	2020-VIII-6	InnerMongolia	101.0752	41.9647	929m		√
EBA03-20111522	Leptopodomorpha	Saldoidea	Saldidae	*Saldula* sp. 3	northwest	temperate zone	middle altitude	2020-VII-29	Ningxia	105.9166	38.7488	1937m	√	√
EBA04-20112107	Leptopodomorpha	Saldoidea	Saldidae	*Saldula* sp. 4	northwest	plateau temperate zone	sub-high altitude	2020-VIII-12	Tibet	95.5233	29.7017	2620m	√	√
EBA05-21101902	Leptopodomorpha	Saldoidea	Saldidae	*Saldula* sp. 5	southeast	mid-subtropical zone	middle altitude	2021-IV-26	Sichuan	102.3873	27.0843	1541	√	√
EBA06-21101702	Leptopodomorpha	Saldoidea	Saldidae	*Saldula* sp. 6	southeast	mid-subtropical zone	middle altitude	2021-IV-28	Sichuan	102.2780	27.0789	1564	√	√
EBA07-20111523	Leptopodomorpha	Saldoidea	Saldidae	*Halosalda halophila*	northwest	temperate zone	low altitude	2020-VIII-5	InnerMongolia	101.2294	42.3241	870m		√
EBA08-20112109	Leptopodomorpha	Saldoidea	Saldidae	*Calacanthia angulosa*	northwest	plateau temperate zone	high altitude	2020-VIII-15	Tibet	97.0517	29.3274	4618m		√
EBA09-20112113	Leptopodomorpha	Saldoidea	Saldidae	*Chartoscrirta* sp.	southeast	mid-subtropical zone	middle altitude	2020-VIII-23	Yunnan	98.6000	28.0158	1781m		√
EBA10-20112112	Leptopodomorpha	Saldoidea	Saldidae	*Salda* sp.	northwest	mid-subtropical zone	sub-high altitude	2020-VIII-19	Tibet	97.7742	28.6514	3945m	√	√
EBA11-21101810	Leptopodomorpha	Saldoidea	Saldidae	*Saldoida armata*	southeast	mid-subtropical zone	low altitude	2021-VII-15~17	Chongqing	106.4206	28.6753	927		√
FAA01-20112324	Cimicomorpha	Reduvioidea	Reduviidae	*Cnizocoris* sp.	northwest	temperate zone	middle altitude	2020.7.31	Ningxia	105.9402	38.7397	1730m	√	√
FAA02-19100906	Cimicomorpha	Reduvioidea	Reduviidae	*Sycanus collaris*	southeast	south subtropical zone	low altitude	2019.9.13	Guangxi	106.7081	22.1992	100m		√
FAA03-19102405	Cimicomorpha	Reduvioidea	Reduviidae	*Isyndus sinicus*	southeast	marginal tropical zone	low altitude	2020.7.30	Yunnan	99.6822	22.1360	940m		√
FAA04-19102507	Cimicomorpha	Reduvioidea	Reduviidae	*Pirates* sp.	southeast	marginal tropical zone	low altitude	2019.8.5	Yunnan	99.0739	23.2739	566m		√
FAA05-20112322	Cimicomorpha	Reduvioidea	Reduviidae	*Pirates fulvescens*	northwest	warm temperate zone	low altitude	2020.8.21	Shanxi	112.4408	35.2736	658m		√
FBA01-20112306	Cimicomorpha	Miroidea	Miridae	*Apolygus ornatus*	northwest	temperate zone	middle altitude	2020.8.31	Ningxia	105.9402	38.7397	1730m		√
FBA02-20112309	Cimicomorpha	Miroidea	Miridae	*Cyphodemidea saundersi*	northwest	warm temperate zone	sub-high altitude	2020.7.27	Ningxia	106.3508	35.3173	2191m	√	√
FBA03-21062403	Cimicomorpha	Miroidea	Miridae	*Deraeocoris* sp.	southeast	mid-subtropical zone	middle altitude	2021.5.3	Sichuan	101.4635	26.8075	1333m	√	√
FBA04-21062404	Cimicomorpha	Miroidea	Miridae	*Cyrtorhinus lividipennis*	southeast	marginal tropical zone	low altitude	2021.5.18	Yunnan	101.2117	21.3691	579m	√	√
FBA05-20112122	Cimicomorpha	Miroidea	Miridae	*Eurystylus coelestialium*	northwest	warm temperate zone	low altitude	2020.8.22	Shanxi	112.4714	35.2428	564m		√

FBA06-20112123	Cimicomorpha	Miroidea	Miridae	*Adelphocoris lineolatus*	northwest	temperate zone	middle altitude	2020.8.11	InnerMongolia	111.5458	40.8344	1089m	√	√
FBA07-20112301	Cimicomorpha	Miroidea	Miridae	*Ectmetopterus* sp.	northwest	warm temperate zone	low altitude	2020.8.21	Shanxi	112.4408	35.2736	658m		√
FBA08-20112304	Cimicomorpha	Miroidea	Miridae	*Deraeocoris punctulatus*	northwest	temperate zone	middle altitude	2020.8.11	InnerMongolia	111.5458	40.8344	1089m	√	√
FBB01-19112605	Cimicomorpha	Miroidea	Tingidae	*Stephanitis* sp.	southeast	marginal tropical zone	middle altitude	2019.7.28	Yunnan	100.5865	21.8372	1878m		√
FBB02-20112315	Cimicomorpha	Miroidea	Tingidae	*Monosteira* sp.	northwest	temperate zone	low altitude	2020.8.4	InnerMongolia	101.0861	41.9522	888m		√
FBB03-20112321	Cimicomorpha	Miroidea	Tingidae	*Physatocheila* sp.	northwest	temperate zone	middle altitude	2020.7.31	Ningxia	105.9402	38.7397	1730m		√
FBB04-21062412	Cimicomorpha	Miroidea	Tingidae	*Tingis* sp.	southeast	mid-subtropical zone	middle altitude	2021.5.6	Yunnan	102.1383	23.6538	1839m	√	√
FCA01-19102801	Cimicomorpha	Naboidea	Nabidae	*Nabis pallida*	southeast	north subtropics zone	low altitude	2019.9.29	Tianjin	117.4481	38.8275	0m		√
FCA02-20112320	Cimicomorpha	Naboidea	Nabidae	*Himacerus mussooriensis*	northwest	warm temperate zone	middle altitude	2020.7.28	Ningxia	106.4306	35.5589	1762m		√
FCA03-20112413	Cimicomorpha	Naboidea	Nabidae	*Halonabis sareptanus*	northwest	temperate zone	low altitude	2020.8.6	InnerMongolia	101.0752	41.9648	929m	√	√
FDA01-20112319	Cimicomorpha	Cimicoidea	Anthocoridae	*Elatophilus* sp.	northwest	temperate zone	middle altitude	2020.8.1	Ningxia	105.9166	38.7488	1937m	√	√
FDA02-20070602	Cimicomorpha	Cimicoidea	Anthocoridae	*Lippomanus* sp.	southeast	south subtropical zone	low altitude	2020.5.20	Guangxi	105.7911	23.0117	225m		√
FDA03-20112312	Cimicomorpha	Cimicoidea	Anthocoridae	*Orius* sp.	northwest	temperate zone	middle altitude	2020.8.12	InnerMongolia	111.5734	40.8473	1113m	√	√
FDA04-20112313	Cimicomorpha	Cimicoidea	Anthocoridae	*Anthocoris pilosus*	northwest	temperate zone	sub-high altitude	2020.7.30	Ningxia	105.9113	38.7360	2283m	√	√
GAA01-19102303	Pentatomomorpha	Aradoidea	Aradidae	*Mezira montana*	southeast	marginal tropical zone	middle altitude	2019.7.26	Yunnan	100.5014	21.6257	1188m	√	√
GAA02-19102305	Pentatomomorpha	Aradoidea	Aradidae	*Mezira hsiaoi*	southeast	marginal tropical zone	middle altitude	2019.7.26	Yunnan	100.5014	21.6257	1188m	√	√
GBA01-19092804	Pentatomomorpha	Pentatomoidea	Acanthosomatidae	*Acanthosoma distinctum*	southeast	mid-subtropical zone	middle altitude	2019.9.16	Guangxi	109.1606	25.4217	1303m	√	√
GBA02-20112414	Pentatomomorpha	Pentatomoidea	Acanthosomatidae	*Sastragala esakii*	northwest	warm temperate zone	low altitude	2020.8.21	Shanxi	112.4714	35.2428	564m	√	√
GBB01-19102501	Pentatomomorpha	Pentatomoidea	Cydnidae	*Geotomus convexus*	southeast	south subtropical zone	low altitude	2019.8.1	Yunnan	100.3721	22.6626	896m		√
GBB02-20112416	Pentatomomorpha	Pentatomoidea	Cydnidae	*Adomerus notatus*	northwest	temperate zone	middle altitude	2020.8.11	InnerMongolia	111.5458	40.8344	1089m	√	√
GBC01-19102201	Pentatomomorpha	Pentatomoidea	Dinidoridae	*Coridius chinensis*	southeast	south subtropical zone	low altitude	2019.7.18	Yunnan	101.0205	22.5837	893m		√
GBC02-19102401	Pentatomomorpha	Pentatomoidea	Dinidoridae	*Cyclopelta obscura*	southeast	marginal tropical zone	low altitude	2019.7.23	Yunnan	100.6864	21.6004	744m		√
GBD01-19101408	Pentatomomorpha	Pentatomoidea	Pentatomidae	*Megarrhamphus truncatus*	southeast	mid-subtropical zone	low altitude	2019.9.18	Guangdong	114.2602	24.7154	397m		√
GBD02-19102408	Pentatomomorpha	Pentatomoidea	Pentatomidae	*Cazira* sp.	southeast	marginal tropical zone	middle altitude	2019.7.28	Yunnan	100.5865	21.8372	1878m		√
GBD03-20112406	Pentatomomorpha	Pentatomoidea	Pentatomidae	*Brachycerocoris camelus*	northwest	warm temperate zone	low altitude	2020.8.22	Shanxi	112.4714	35.2427	514m	√	√
GBE01-19102506	Pentatomomorpha	Pentatomoidea	Plataspidae	*Megacopta cribraria*	southeast	marginal tropical zone	low altitude	2019.7.20	Yunnan	101.2092	21.9662	580m		√
GBF01-19082903	Pentatomomorpha	Pentatomoidea	Saileriolidae	*Bannacoris arboreus*	southeast	mid-subtropical zone	middle altitude	2019.8.16	Yunnan	98.4786	25.0178	1896m	√	√
GBG01-19092307	Pentatomomorpha	Pentatomoidea	Scutelleridae	*Poecilocoris latus*	southeast	mid-subtropical zone	low altitude	2019.9.18	Guangdong	114.2602	24.7154	397m		√
GBG02-20112407	Pentatomomorpha	Pentatomoidea	Scutelleridae	*Hyperoncus lateritius*	northwest	mid-subtropical zone	middle altitude	2020.8.18	Tibet	97.0536	28.3569	1353m	√	√
GBH01-19102203	Pentatomomorpha	Pentatomoidea	Tessaratomidae	*Pycanum ochraceum*	southeast	marginal tropical zone	middle altitude	2019.7.27	Yunnan	100.5907	21.8529	1613m		√
GBH02-19102204	Pentatomomorpha	Pentatomoidea	Tessaratomidae	*Eurostus ochraceus*	southeast	mid-subtropical zone	middle altitude	2019.8.16	Yunnan	98.4786	25.0178	1896m		√

GBI01-19092801	Pentatomomorpha	Pentatomoidea	Urostylididae	*Urolabida histrionica*	southeast	marginal tropical zone	middle altitude	2019.7.24	Yunnan	100.3525	21.5734	1222m	√	√
GBI02-20111303	Pentatomomorpha	Pentatomoidea	Urostylididae	*Urochela quadrinotata*	northwest	north subtropics zone	middle altitude	2020.10.18	Shaanxi	108.9048	33.7336	1261m	√	√
GCA01-19082905	Pentatomomorpha	Pyrrhocoroidea	Largidae	*Physopelta gutta*	southeast	south subtropical zone	low altitude	2019.7.16	Guangxi	108.2694	22.1334	2m	√	√
GCA02-19090912	Pentatomomorpha	Pyrrhocoroidea	Largidae	*Macroceroea grandis*	southeast	marginal tropical zone	low altitude	2019.7.20	Yunnan	101.2517	21.9350	569m	√	√
GCA03-19091704	Pentatomomorpha	Pyrrhocoroidea	Largidae	*Iphita limbata*	southeast	marginal tropical zone	low altitude	2019.7.19	Yunnan	101.2517	21.9350	893m	√	√
GCA04-19102406	Pentatomomorpha	Pyrrhocoroidea	Largidae	*Physopelta quadriguttata*	southeast	marginal tropical zone	middle altitude	2019.7.28	Yunnan	100.5865	21.8372	1878m		√
GCB01-19090406	Pentatomomorpha	Pyrrhocoroidea	Pyrrhocoridae	*Dysdercus cingulatus*	southeast	marginal tropical zone	low altitude	2019.8.8	Yunnan	98.9501	23.5120	440m	√	√
GCB02-19090901	Pentatomomorpha	Pyrrhocoroidea	Pyrrhocoridae	*Antilochus nigripes*	southeast	south subtropical zone	middle altitude	2019.7.31	Yunnan	100.3721	22.6626	1203m		√
GCB03-20070607	Pentatomomorpha	Pyrrhocoroidea	Pyrrhocoridae	*Euscopus fuscus*	southeast	marginal tropical zone	middle altitude	2020.5.7	Yunnan	102.2517	22.4997	1140m		√
GCB04-20112409	Pentatomomorpha	Pyrrhocoroidea	Pyrrhocoridae	*Pyrrhocoris sinuaticollis*	northwest	warm temperate zone	low altitude	2020.8.25	Shanxi	112.8898	35.4913	761m	√	√
GDA01-19100907	Pentatomomorpha	Coreoidea	Alydidae	*Riptortus linearis*	southeast	marginal tropical zone	low altitude	2019.9.9	Yunnan	101.2779	21.2576	633m		√
GDA02-20112419	Pentatomomorpha	Coreoidea	Alydidae	*Megalotomus junceus*	northwest	warm temperate zone	middle altitude	2020.8.24	Shanxi	112.8376	35.5632	1001m	√	√
GDB01-19090405	Pentatomomorpha	Coreoidea	Coreidae	*Cloreunus modestus*	southeast	marginal tropical zone	low altitude	2019.8.5	Yunnan	99.0739	23.2739	566m		√
GDB02-19102105	Pentatomomorpha	Coreoidea	Coreidae	*Homoeocerus striicornis*	southeast	south subtropical zone	low altitude	2019.9.13	Guangxi	106.7081	22.1992	100m		√
GDB03-19102802	Pentatomomorpha	Coreoidea	Coreidae	*Hydaropsis longirostris*	southeast	south subtropical zone	low altitude	2019.9.3	Yunnan	98.0705	24.0898	880m		√
GDC01-20070406	Pentatomomorpha	Coreoidea	Rhopalidae	*Leptocoris vicinus*	southeast	south subtropical zone	low altitude	2020.7.2	Guangdong	113.2903	23.0958	4m		√
GDC02-20112412	Pentatomomorpha	Coreoidea	Rhopalidae	*Corizus tetraspilus*	northwest	temperate zone	middle altitude	2020.8.4	InnerMongolia	111.5458	40.8344	1089m		√
GEA01-19102904	Pentatomomorpha	Lygaeoidea	Berytidae	*Metatropis brevirostris*	southeast	south subtropical zone	low altitude	2018.8.28	Yunnan	97.5893	23.9689	950m		√
GEB01-20111516	Pentatomomorpha	Lygaeoidea	Geocoridae	*Geocoris grylloides*	northwest	temperate zone	middle altitude	2020.8.11	InnerMongolia	111.5458	40.8344	1089m	√	√
GEB02-20112422	Pentatomomorpha	Lygaeoidea	Geocoridae	*Henestaris oschanini*	northwest	temperate zone	low altitude	2020.8.8	InnerMongolia	108.7692	40.8287	996m	√	√
GEC01-19091711	Pentatomomorpha	Lygaeoidea	Heterogastridae	*Dinomachus sikhimensis*	southeast	marginal tropical zone	low altitude	2019.7.22	Yunnan	101.2092	21.9662	580m		√
GED01-20111511	Pentatomomorpha	Lygaeoidea	Lygaeidae	*Lygaeus hanseni*	northwest	temperate zone	middle altitude	2020.8.1	Ningxia	105.9166	38.7488	1937m		√
GEE01-19091705	Pentatomomorpha	Lygaeoidea	Malcidae	*Malcus sinicus*	southeast	mid-subtropical zone	middle altitude	2019.8.15	Yunnan	98.7944	25.2949	1560m	√	√
GEF01-19103004	Pentatomomorpha	Lygaeoidea	Rhyparochromidae	*Elasmolomus squalidus*	southeast	south subtropical zone	low altitude	2019.8.1	Yunnan	100.3721	22.6626	896m		√

Table S2. The statistics for sequences and features after quality control.

	**16S**	**ITS**
Number of samples	204	71
Number of features	3,063	1,173
Total frequency	1,019,323	387,572
Minimum frequency	242	901
1st quartile	3,218	3,652
Median frequency	4,788	4,562
3rd quartile	5,907	6,819
Maximum frequency	10,914	13,324
Mean frequency	4,997	5,459

Table S3. Adjusted *p* values for pairwise comparisons of bacterial community compositions.

16S-Alpha diversity		Faith's PD			Pielou's evenness			Observed features			Shannon entropy		
Group 1	Group 2	LSD	KW	PA	LSD	KW	PA	LSD	KW	PA	LSD	KW	PA
**Infraorder**													
Dipsocoromorpha	Enicocephalomorpha	0.637	0.686	0.953	0.863	0.878	0.963	0.903	0.808	0.903	1.017	0.917	0.988
Dipsocoromorpha	Gerromorpha	0.832	0.918	0.953	1.087	0.930	0.999	0.832	0.808	0.903	1.041	0.860	0.988
Dipsocoromorpha	Nepomorpha	0.828	0.686	0.953	1.093	0.878	0.999	0.856	0.808	0.903	1.093	0.956	0.988
Dipsocoromorpha	Leptopodomorpha	0.815	0.918	0.953	1.044	0.930	0.999	0.788	0.808	0.903	1.040	0.956	0.988
Dipsocoromorpha	Cimicomorpha	0.683	0.723	0.953	0.943	0.930	0.963	1.176	0.808	0.903	1.061	0.860	0.988
Dipsocoromorpha	Pentatomomorpha	0.575	0.686	0.953	0.845	0.383	0.963	1.004	0.953	0.964	0.876	0.789	0.988
Enicocephalomorpha	Gerromorpha	0.548	0.686	0.953	0.956	0.878	0.963	0.902	0.808	0.903	1.091	0.956	0.988
Enicocephalomorpha	Nepomorpha	0.519	0.505	0.953	0.961	0.878	0.963	0.787	0.808	0.903	1.038	0.956	0.988
Enicocephalomorpha	Leptopodomorpha	0.650	0.686	0.953	0.909	0.878	0.963	0.883	0.808	0.903	0.994	1.000	0.988
Enicocephalomorpha	Cimicomorpha	0.807	0.759	0.953	0.825	0.878	0.963	0.952	0.885	0.964	1.050	0.860	0.988
Enicocephalomorpha	Pentatomomorpha	0.852	0.949	0.953	1.046	0.927	0.999	1.061	0.808	0.903	1.077	0.860	0.988
Gerromorpha	Nepomorpha	0.774	0.544	0.953	0.846	0.878	0.999	1.106	0.808	0.903	0.784	0.696	0.988
Gerromorpha	Leptopodomorpha	0.746	0.686	0.953	0.941	0.930	0.999	0.998	0.909	0.964	0.992	0.860	0.988
Gerromorpha	Cimicomorpha	0.247	0.472	0.953	0.775	0.878	0.963	1.146	0.808	0.903	0.943	0.860	0.988
Gerromorpha	Pentatomomorpha	**0.032**	0.085	0.953	0.179	**0.032**	0.963	1.169	0.808	0.903	0.126	0.114	0.988
Nepomorpha	Leptopodomorpha	0.697	0.505	0.953	0.996	0.878	0.999	0.994	0.808	0.903	0.997	0.860	0.988
Nepomorpha	Cimicomorpha	0.084	**0.044**	0.953	0.680	0.878	0.963	1.008	0.808	0.903	0.518	0.461	0.988
Nepomorpha	Pentatomomorpha	**0.000**	**0.002**	0.953	0.308	0.103	0.963	0.879	0.808	0.903	0.730	0.486	0.988
Leptopodomorpha	Cimicomorpha	0.636	0.686	0.953	0.938	0.878	0.963	0.759	0.808	0.903	0.792	0.789	0.988
Leptopodomorpha	Pentatomomorpha	0.585	0.507	0.953	0.761	0.123	0.963	1.126	0.808	0.903	0.659	0.403	0.988
Cimicomorpha	Pentatomomorpha	0.829	0.778	0.953	0.105	0.093	0.963	1.092	0.808	0.903	0.105	0.114	0.988
**Area**													
Northwest	Southeast		0.377	0.766		**0.000**	0.080		**0.000**	0.108		**0.000**	0.062

LSD: LSD post-hoc test. KW: Kruskal-wallis test. PA: Phylogenetic ANOVA analysis.

Significant values (adjusted $p < 0.05$) are shown in bold.

Table S4. Statistical analyses for the results of beta diversity based on (un)weighted UniFrac distance and PERMANOVA test.

16S & ITS-Beta diversity		Unweighted UniFrac distance				Weighted UniFrac distance			
		16S		ITS		16S		ITS	
Group 1	Group 2	p-value	q-value	p-value	q-value	p-value	q-value	p-value	q-value
**Infraorder**									
Dipsocoromorpha	Enicocephalomorpha	0.268	0.296	1.000	1.000	0.279	0.410	1.000	1.000
Dipsocoromorpha	Gerromorpha	0.550	0.550	0.870	0.938	0.312	0.410	0.816	0.902
Dipsocoromorpha	Nepomorpha	**0.019**	**0.044**	0.802	0.938	0.059	0.138	0.805	0.902
Dipsocoromorpha	Leptopodomorpha	0.134	0.212	0.782	0.938	0.152	0.290	0.517	0.797
Dipsocoromorpha	Cimicomorpha	0.196	0.242	0.124	0.372	0.252	0.410	0.113	0.396
Dipsocoromorpha	Pentatomomorpha	0.177	0.232	0.652	0.931	0.619	0.650	0.666	0.823
Enicocephalomorpha	Gerromorpha	0.134	0.212	0.852	0.938	0.581	0.642	0.931	0.978
Enicocephalomorpha	Nepomorpha	**0.009**	**0.024**	0.665	0.931	0.523	0.610	0.583	0.797
Enicocephalomorpha	Leptopodomorpha	0.165	0.231	0.893	0.938	0.381	0.471	0.582	0.797
Enicocephalomorpha	Cimicomorpha	0.232	0.271	0.194	0.509	0.303	0.410	0.428	0.797
Enicocephalomorpha	Pentatomomorpha	0.125	0.212	0.318	0.668	0.294	0.410	0.539	0.797
Gerromorpha	Nepomorpha	**0.001**	**0.004**	0.528	0.853	**0.001**	**0.004**	0.164	0.492
Gerromorpha	Leptopodomorpha	**0.004**	**0.014**	0.470	0.853	0.122	0.256	0.607	0.797
Gerromorpha	Cimicomorpha	**0.021**	**0.044**	**0.020**	0.137	**0.039**	0.102	**0.040**	0.210
Gerromorpha	Pentatomomorpha	**0.001**	**0.004**	0.094	0.329	**0.001**	**0.004**	0.240	0.504
Nepomorpha	Leptopodomorpha	**0.001**	**0.004**	0.511	0.853	**0.001**	**0.004**	0.217	0.504
Nepomorpha	Cimicomorpha	**0.001**	**0.004**	**0.004**	0.084	**0.001**	**0.004**	**0.004**	0.084
Nepomorpha	Pentatomomorpha	**0.001**	**0.004**	**0.026**	0.137	**0.001**	**0.004**	**0.026**	0.196
Leptopodomorpha	Cimicomorpha	0.347	0.364	**0.015**	0.137	0.761	0.761	0.100	0.396
Leptopodomorpha	Pentatomomorpha	**0.007**	**0.021**	0.274	0.639	**0.006**	**0.021**	0.207	0.504
Cimicomorpha	Pentatomomorpha	0.141	0.212	0.051	0.214	**0.036**	0.102	**0.028**	0.196
**Area**									
Northwest	Southeast	**0.003**	**0.003**	**0.001**	**0.001**	**0.015**	**0.015**	**0.001**	**0.001**

Significant values ($p < 0.05$ and $q < 0.05$) are shown in bold.

Table S5. Adjusted *p* values for pairwise comparisons of fungal community compositions.

ITS-Alpha diversity		Faith's PD			Pielou's evenness			Observed features			Shannon entropy		
**Group 1**	**Group 2**	**LSD**	**KW**	**PA**	**LSD**	**KW**	**PA**	**LSD**	**KW**	**PA**	**LSD**	**KW**	**PA**
**Infraorder**													
Dipsocoromorpha	Enicocephalomorpha		1.000			0.522			1.000			1.000	
Dipsocoromorpha	Gerromorpha	0.972	1.000	0.964	0.513	0.594	0.618	0.864	1.000	0.863	0.762	0.978	0.779
Dipsocoromorpha	Nepomorpha	1.129	0.987	0.964	0.845	0.946	0.827	0.984	1.000	0.863	0.964	1.000	0.962
Dipsocoromorpha	Leptopodomorpha	1.235	0.987	0.964	0.734	0.837	0.706	0.903	1.000	0.863	0.955	0.978	0.962
Dipsocoromorpha	Cimicomorpha	1.159	0.987	0.964	0.488	0.946	0.645	0.967	1.000	0.977	0.702	0.978	0.814
Dipsocoromorpha	Pentatomomorpha	1.198	0.987	0.964	0.360	0.522	0.413	0.959	1.000	0.863	0.505	0.978	0.660
Enicocephalomorpha	Gerromorpha		0.987			0.946			1.000			0.978	
Enicocephalomorpha	Nepomorpha		0.987			0.522			1.000			0.978	
Enicocephalomorpha	Leptopodomorpha		0.987			0.522			1.000			0.978	
Enicocephalomorpha	Cimicomorpha		0.710			0.522			1.000			0.978	
Enicocephalomorpha	Pentatomomorpha		0.948			1.000			1.000			0.978	
Gerromorpha	Nepomorpha	0.975	0.987	0.964	0.348	0.522	0.495	0.895	1.000	0.863	0.495	0.978	0.660
Gerromorpha	Leptopodomorpha	1.054	0.987	0.964	0.569	0.522	0.645	0.879	1.000	0.863	0.597	0.978	0.779
Gerromorpha	Cimicomorpha	0.656	0.710	0.964	0.819	0.594	0.895	1.258	1.000	0.863	0.900	0.978	0.962
Gerromorpha	Pentatomomorpha	0.575	0.710	0.964	0.339	0.594	0.543	0.750	0.604	0.863	0.604	0.978	0.675
Nepomorpha	Leptopodomorpha	0.980	1.000	0.972	0.695	0.871	0.658	0.927	1.000	0.875	0.931	0.978	0.864
Nepomorpha	Cimicomorpha	1.785	0.710	0.964	0.375	0.522	0.645	0.773	0.537	0.863	0.540	0.978	0.779
Nepomorpha	Pentatomomorpha	1.140	0.710	0.964	**0.030**	**0.026**	0.255	0.525	0.537	0.863	0.060	0.229	0.285
Leptopodomorpha	Cimicomorpha	0.770	0.710	0.964	0.485	0.837	0.658	0.987	0.537	0.863	0.546	0.978	0.779
Leptopodomorpha	Pentatomomorpha	0.603	0.710	0.964	0.188	0.079	0.425	0.698	0.537	0.863	0.210	0.323	0.660
Cimicomorpha	Pentatomomorpha	1.255	0.987	0.964	0.439	0.120	0.645	1.093	1.000	0.863	0.561	0.810	0.779
**Area**													
Northwest	Southeast		**0.000**	**0.001**		0.160	0.256		**0.000**	**0.002**		**0.002**	0.065

LSD: LSD post-hoc test. KW: Kruskal-wallis test. PA: Phylogenetic ANOVA analysis.

Significant values (adjusted $p < 0.05$) are shown in bold.

Table S6. Primer sequences of PCR amplification for 16S rRNA, ITS, CO1, CO2, 18S rRNA and 28S rRNA genes.

Target gene	Primer name	Primer sequence (5'→3')	Length	Fwd./Rev.
16S	27F	AGAGTTTGATCCTGGCTCAG	20	Fwd.
16S	1492R	TACGGCTACCTTGTTACGACTT	22	Rev.
ITS	ITS1	CTTGGTCATTTAGAGGAAGTAA	22	Fwd.
ITS	ITS4	TCCTCCGCTTATTGATATGC	20	Rev.
18S	18S-Ns1-F	GTAGTCATATGCTTGTCTC	19	Fwd.
18S	18SP3-R	GGTTAGAACTAGGGCGGTATCT	22	Rev.
18S	18SP5-F	CAAGAACGAAAGTTAGAGGT	20	Fwd.
18S	18S-Ns8-R	TCCGCAGGTTCACCTACGGA	20	Rev.
28S	28S-SS7-AF	GCGGAGGAAAAGAAACTAAC	20	Fwd.
28S	28S-XS2-AR	GGCATAGTTCACCATCTTTCG	21	Rev.
28S	28S-DF1-BF	ATCCGACCCGTCTTGAAACAC	21	Fwd.
28S	28S-FD1-BR	TGCTACTACCACCAAGATCTG	21	Rev.
28S	28S-EE-CF	ATCCGCTAAGGAGTGTGTAA	20	Fwd.
28S	28S-GG-CR	CCGACTTCCCTTACCTACATT	21	Rev.
28S	28S-OP-DF	TAGGAGGGAGATAGGGTTTG	20	Fwd.
28S	28S-Pob-DR	TACCGCCCCAGTCAAACTCC	20	Rev.
28S	28S-UZ-EF	AGGTGTAGCATAAGTGGGAG	20	Fwd.
28S	28S-ZU-ER	TTCGGTCTTAGAGGCGTTCAG	21	Rev.
28S	28S-cb4-FF	CGGCTCTTCCTATCATTGCG	20	Fwd.
28S	28S-kb4-FR	CAGCGTGGCAACTGCTCTCC	20	Rev.
CO1	1490	GGTCAACAAATCATAAAGATATTGG	25	Fwd.
CO1	2198	TAAACTTCAGGGTGACCAAAAAATCA	26	Rev.
CO1	C1-J1709	AATTGGWGGWTTYGGAAAYTG	21	Fwd.
CO1	C1-N2776	GGTAATCAGAGTATCGWCGNGG	22	Rev.
CO2	J3043	GGCAGATTAGTGYAATGRATTTAA	24	Fwd.
CO2	N3796	ACTATTAGATGGTTTAAGAG	20	Rev.

Table S7. The species selected as out groups in the reconstruction of phylogenetic relationships.

Order	Suborder	Superfamily: family	Species	CO1	CO2	18S	28S
Hemiptera	Sternorrhyncha	Aphidoidea: Aphididae	*Rhopalosiphum padi*	KT447631	KT447631	AF487718	
Hemiptera	Sternorrhyncha	Psylloidea: Liviidae	*Camarotoscena* sp. YW-2014			KJ461224	KJ461307
Hemiptera	Sternorrhyncha	Psylloidea: Triozidae	*Trioza erytreae*	NC_038142	NC_038142		
Hemiptera	Sternorrhyncha	Phylloxeroidea: Adelgidae	*Adelges tsugae*	MT263947	MT263947	KT199045	
Hemiptera	Auchenorrhyncha	Cercopoidea: Aphrophoridae	*Philaenus spumarius*	AY630340	AY630340	U06480	AY744813
Hemiptera	Auchenorrhyncha	Cicadoidea: Cicadidae	*Cicadetta abscondita*	MW123088	MW123088	MN147807	
Hemiptera	Auchenorrhyncha	Delphacidae	*Nilaparvata lugens*	JX880069	JX880069	JF773148	JX556804
Hemiptera	Auchenorrhyncha	Fulgoridae	*Laternaria candelaria*	FJ006724	FJ006724	JX556774	JX556813
Hemiptera	Auchenorrhyncha	Flatidae	*Geisha distinctissima*	FJ230961	FJ230961	JX556750	
Hemiptera	Coleorrhyncha	Peloridiidae	*Xenophyes cascus*	JF323862	JF323862	KJ461238	KJ461168
Hemiptera	Coleorrhyncha	Peloridiidae	*Peloridora minuta*	JQ739183	JQ739183	HM017319	HM017427
Hemiptera	Coleorrhyncha	Peloridiidae	*Hemiodoecus leai*	KM035420	KM035420		HF547832
Hemiptera	Coleorrhyncha	Peloridiidae	*Hackeriella veitchi*	GQ884145	GQ884145	AF004766	HF547836

Table S8. The statistics for CO1, CO2, 18S rRNA and 28S rRNA genes.

Taxon	Number of genes	Coverage of genes	Number of nucleotide sites	Coverage of nucleotide sites	CO1	CO2	18S	28S
Cryptostemma sp.	4	100.0%	5913	76.0%	598	450	1813	3052
Cryptostemma sp.	4	100.0%	5889	75.7%	574	450	1813	3052
Cryptostemma sp.	4	100.0%	5913	76.0%	598	450	1813	3052
Ceratocombus sp. 1	3	75.0%	4761	61.2%	434	444		3883
Ceratocombus sp. 2	1	25.0%	2573	33.1%				2573
Ceratocombus sp. 3	3	75.0%	6496	83.5%	802		1791	3903
Kokeshia xiei	4	100.0%	6930	89.1%	802	450	1801	3877
Aenictopecheinae sp.	3	75.0%	6460	83.1%	798		1780	3882
Hoplitocoris lewisi	2	50.0%	4053	52.1%	432			3621
Mesovelia vittigera	4	100.0%	6910	88.8%	802	450	1802	3856
Mesovelia sp.	4	100.0%	6580	84.6%	432	450	1796	3902
Mesovelia thermalis	2	50.0%	4291	55.2%	428			3863
Mesovelia vittigera	4	100.0%	6530	84.0%	420	450	1802	3858
Mesovelia vittigera	4	100.0%	6530	84.0%	420	450	1802	3858
Timasius sp. 1	3	75.0%	2542	32.7%	430	450		1662
Timasius sp. 2	4	100.0%	5575	71.7%	802	450	1807	2516
Hebrus sp. 1	4	100.0%	6410	82.4%	432	450	1787	3741
Hebrus sp. 2	4	100.0%	6919	89.0%	802	450	1787	3880
Hebrus sp. 3	4	100.0%	6549	84.2%	432	450	1788	3879
Hyrcanus sp.	3	75.0%	2674	34.4%	434	450		1790
Hydrometra sp. 1	3	75.0%	3691	47.5%	798		1021	1872
Hydrometra sp. 2	3	75.0%	4312	55.4%	800		1838	1674
Hydrometra greeni	4	100.0%	6259	80.5%	802	450	1824	3183
Hydrometra greeni	4	100.0%	6257	80.4%	802	450	1824	3181
Microvelia sp. 5	4	100.0%	6785	87.2%	788	384	1748	3865
Perittopus crinalis	3	75.0%	6433	82.7%	798		1782	3853
Perittopus asiaticus	4	100.0%	6899	88.7%	786	450	1788	3875

Microvelia (Picaultia) douglasi	3	75.0%	6263	80.5%	672		1782	3809
Microvelia (*Cloacovelia*) sp.	4	100.0%	6897	88.7%	794	450	1782	3871
Microvelia (*Picaultia*) sp.	3	75.0%	4975	64.0%	650	446		3879
Microvelia sp. 1	4	100.0%	6656	85.6%	580	414	1778	3884
Microvelia sp. 2	4	100.0%	6754	86.8%	648	450	1782	3874
Microvelia kyushuensis	4	100.0%	6921	89.0%	802	450	1783	3886
Microvelia sp. 3	4	100.0%	6556	84.3%	432	450	1781	3893
Microvelia sp. 4	4	100.0%	6918	88.9%	802	450	1782	3884
Baptista sp. 1	3	75.0%	6505	83.6%	802		1818	3885
Baptista sp. 2	2	50.0%	4675	60.1%	800			3875
Baptista sp. 3	3	75.0%	6458	83.0%	802		1781	3875
Rhagovelia sp.1	3	75.0%	5777	74.3%	802		1791	3184
Rhagovelia sp.2	3	75.0%	5801	74.6%	802		1791	3208
Rhagovelia sp.3	3	75.0%	5816	74.8%	802		1791	3223
Halovelia sp.	4	100.0%	6231	80.1%	802	450	1796	3183
Gerridae sp.	4	100.0%	6579	84.6%	432	446	1792	3909
Amemboa sp.1	4	100.0%	6933	89.1%	802	450	1787	3894
Amemboa sp.2	3	75.0%	6485	83.4%	802		1787	3896
Ptilomera tigrina	4	100.0%	6820	87.7%	786	374	1787	3873
Ptilomera sp.	3	75.0%	6468	83.2%	800		1786	3882
Potamometra sp.1	3	75.0%	6455	83.0%	796		1783	3876
Potamometra sp.2	3	75.0%	6459	83.0%	800		1783	3876
Eotrechus siamensis	3	75.0%	6473	83.2%	802		1788	3883
Limnometra sp.	3	75.0%	6474	83.2%	802		1787	3885
Aquarius paludum	4	100.0%	6929	89.1%	802	450	1787	3890
Aquarius sp.	4	100.0%	6920	89.0%	798	450	1787	3885
Aquarius paludum	4	100.0%	6929	89.1%	802	450	1787	3890
Gerris (Macrogerris) tigrinus	3	75.0%	6471	83.2%	798		1787	3886
Gerris (Macrogerris) gracilicornis	4	100.0%	6921	89.0%	798	450	1787	3886
Gerris sp.	3	75.0%	6327	81.3%	650		1787	3890
Limnogonus nitidus	4	100.0%	6928	89.1%	802	446	1786	3894

Limnogonus fossarum fossarum	4	100.0%	6922	89.0%	802	450	1787	3883
Limnogonus fossarum fossarum	4	100.0%	6930	89.1%	802	450	1788	3890
Limnogonus nitidus	4	100.0%	6932	89.1%	802	446	1788	3896
Limnometra matsudai	3	75.0%	6475	83.2%	800		1787	3888
Metrocoris sp.	3	75.0%	6115	78.6%	432		1787	3896
Gigantometra gigas	4	100.0%	6116	78.6%	802	444	1788	3082
Hermatobates lingyangjiaoensis	4	100.0%	5797	74.5%	802	450	1772	2773
Micronecta sp. 1	2	50.0%	4337	55.8%		450		3887
Micronecta sp. 2	4	100.0%	6737	86.6%	610	432	1817	3878
Micronecta sp. 3	3	75.0%	6137	78.9%		450	1817	3870
Micronecta sp. 4	4	100.0%	6941	89.2%	782	450	1817	3892
Micronecta sp. 5	4	100.0%	6948	89.3%	802	450	1814	3882
Micronecta sp. 6	4	100.0%	6928	89.1%	786	440	1818	3884
Micronecta sp. 7	4	100.0%	6948	89.3%	802	450	1814	3882
Micronecta sp. 8	4	100.0%	6949	89.3%	802	450	1819	3878
Micronecta sp. 9	4	100.0%	6948	89.3%	802	446	1816	3884
Cenocorixa sp. 1	4	100.0%	6926	89.0%	800	450	1811	3865
Cenocorixa sp. 2	4	100.0%	6927	89.1%	794	450	1811	3872
Cenocorixa sp. 3	4	100.0%	6923	89.0%	802	450	1795	3876
Cenocorixa sp. 4	3	75.0%	6093	78.3%	430		1810	3853
Sigara sp.1	4	100.0%	6889	88.6%	764	450	1804	3871
Sigara sp.2	4	100.0%	6941	89.2%	802	450	1811	3878
Sigara sp.3	4	100.0%	6618	85.1%	802	152	1808	3856
Callicorixa sp.	4	100.0%	6932	89.1%	802	450	1803	3877
Cymatia sp.	4	100.0%	6925	89.0%	800	450	1804	3871
Ranatra sp. 1	4	100.0%	6306	81.1%	802	432	1820	3252
Ranatra sp. 2	4	100.0%	6768	87.0%	802	446	1817	3703
Ranatra sterea	4	100.0%	5641	72.5%	430	450	1822	2939
Cercotmetus asiaticus	3	75.0%	2954	38.0%	430	448		2076
Laccotrephes japonensis	4	100.0%	6067	78.0%	802	450	1805	3010
Laccotrephes japonensis	4	100.0%	6073	78.1%	802	450	1805	3016

Laccotrephes japonensis	4	100.0%	6073	78.1%	802	450	1805	3016
Diplonychus sp. 1	4	100.0%	5017	64.5%	802	450	1828	1937
Diplonychus sp. 2	4	100.0%	5799	74.6%	802	446	1805	2746
Lethocerus deyrollei	4	100.0%	6600	84.9%	802	450	1807	3541
Nerthra indica	4	100.0%	6904	88.8%	802	450	1819	3833
Nerthra indica	4	100.0%	6904	88.8%	802	450	1819	3833
Nerthra indica	4	100.0%	6904	88.8%	802	450	1819	3833
Nerthra indica	4	100.0%	6904	88.8%	802	450	1819	3833
Ochterus sp. 1	3	75.0%	4765	61.3%	798	450		3517
Ochterus sp. 2	4	100.0%	6981	89.8%	802	450	1815	3914
Ochterus sp. 3	4	100.0%	6978	89.7%	802	450	1814	3912
Notonecta sp. 1	4	100.0%	6927	89.1%	802	450	1814	3861
Notonecta sp. 2	4	100.0%	6924	89.0%	802	450	1809	3863
Notonecta sp. 3	4	100.0%	5746	73.9%	434	450	1619	3243
Notonecta sp. 4	4	100.0%	6927	89.1%	802	450	1814	3861
Notonecta chinensis	4	100.0%	6930	89.1%	802	450	1813	3865
Enithares sp.	3	75.0%	6419	82.5%	802		1752	3865
Anisops sp. 1	4	100.0%	6657	85.6%	492	450	1822	3893
Anisops sp. 2	4	100.0%	6980	89.7%	802	450	1817	3911
Anisops sp. 3	4	100.0%	6982	89.8%	790	450	1816	3926
Anisops kuroiwae	4	100.0%	6972	89.6%	790	450	1822	3910
Helotrephes sp. 1	4	100.0%	4085	52.5%	796	450	64	2775
Helotrephes sp. 2	3	75.0%	5097	65.5%	790	450		3857
Helotrephes sp. 3	4	100.0%	6916	88.9%	802	450	1807	3857
Helotrephes tuberculatus	3	75.0%	5106	65.6%	802	450		3854
Paraplea indistinguenda	4	100.0%	6928	89.1%	802	450	1812	3864
Paraplea liturata	3	75.0%	4744	61.0%	432	450		3862
Gestroiella sp. 1	4	100.0%	6827	87.8%	802	450	1715	3860
Gestroiella sp. 2	4	100.0%	6895	88.6%	802	450	1804	3839
Thurselinus scutellaris	4	100.0%	6362	81.8%	802	450	1807	3303
Thurselinus scutellaris	4	100.0%	5998	77.1%	802	450	1807	2939

Aphelocheirus sp. 1	4	100.0%	6907	88.8%	798	450	1805	3854
Aphelocheirus sp. 2	4	100.0%	6890	88.6%	788	450	1805	3847
Potamocoris sp.	3	75.0%	4287	55.1%	604	448		3235
Valleriola sp.	4	100.0%	3329	42.8%	594	446	769	1520
Valleriola sp.	2	50.0%	3976	51.1%	116			3860
Valleriola javanica	4	100.0%	6575	84.5%	802	446	1751	3576
Leptopus sp.	4	100.0%	6703	86.2%	586	446	1810	3861
Corallocoris xishaensis	4	100.0%	6881	88.5%	800	450	1803	3828
Saldula sp. 1	4	100.0%	6878	88.4%	802	450	1795	3831
Saldula sp. 2	4	100.0%	6872	88.4%	794	450	1794	3834
Saldula sp. 3	4	100.0%	6877	88.4%	802	450	1789	3836
Saldula sp. 4	4	100.0%	6883	88.5%	802	450	1794	3837
Saldula sp. 5	4	100.0%	6679	85.9%	598	450	1794	3837
Saldula sp. 6	4	100.0%	6876	88.4%	800	450	1793	3833
Halosalda halophila	4	100.0%	6878	88.4%	802	450	1790	3836
Calacanthia angulosa	4	100.0%	6872	88.4%	802	450	1786	3834
Chartoscrirta sp.	4	100.0%	6447	82.9%	358	450	1800	3839
Salda sp.	4	100.0%	6844	88.0%	762	450	1800	3832
Saldoida armata	4	100.0%	6712	86.3%	644	450	1799	3819
Cnizocoris sp.	4	100.0%	6646	85.4%	802	450	1818	3576
Sycanus collaris	4	100.0%	6698	86.1%	802	450	1770	3676
Isyndus sinicus	4	100.0%	4919	63.2%	802	450	1810	1857
Pirates sp.	4	100.0%	7037	90.5%	800	450	1821	3966
Pirates fulvescens	4	100.0%	7076	91.0%	802	450	1822	4002
Apolygus ornatus	4	100.0%	6617	85.1%	434	450	1831	3902
Cyphodemidea saundersi	4	100.0%	6985	89.8%	802	450	1832	3901
Deraeocoris sp.	3	75.0%	5252	67.5%	424		1821	3007
Cyrtorhinus lividipennis	2	50.0%	2776	35.7%	796			1980
Eurystylus coelestialium	2	50.0%	4334	55.7%	434			3900
Adelphocoris lineolatus	4	100.0%	6898	88.7%	802	450	1828	3818
Ectmetopterus sp.	3	75.0%	6473	83.2%	800		1817	3856

Deraeocoris punctulatus	4	100.0%	6988	89.8%	802	450	1832	3904
Stephanitis sp.	4	100.0%	6641	85.4%	434	450	1815	3942
Monosteira sp.	4	100.0%	6896	88.7%	662	450	1829	3955
Physatocheila sp.	4	100.0%	7058	90.7%	802	450	1833	3973
Tingis sp.	4	100.0%	4830	62.1%	798	450	1832	1750
Nabis pallida	4	100.0%	6592	84.8%	622	450	1808	3712
Himacerus mussooriensis	4	100.0%	6903	88.8%	800	450	1809	3844
Halonabis sareptanus	4	100.0%	6893	88.6%	800	450	1806	3837
Elatophilus sp.	4	100.0%	6924	89.0%	802	448	1817	3857
Lippomanus sp.	3	75.0%	4715	60.6%	434	450		3831
Orius sp.	4	100.0%	6906	88.8%	780	450	1817	3859
Anthocoris pilosus	4	100.0%	6860	88.2%	802	450	1815	3793
Mezira montana	4	100.0%	6983	89.8%	802	446	1830	3905
Mezira hsiaoi	4	100.0%	6598	84.8%	406	446	1832	3914
Acanthosoma distinctum	4	100.0%	6981	89.8%	802	450	1822	3907
Sastragala esakii	4	100.0%	6941	89.2%	802	450	1795	3894
Geotomus convexus	3	75.0%	6516	83.8%	802		1819	3895
Adomerus notatus	4	100.0%	6938	89.2%	802	450	1799	3887
Coridius chinensis	4	100.0%	6906	88.8%	802	450	1747	3907
Cyclopelta obscura	4	100.0%	6926	89.0%	802	450	1821	3853
Megarrhamphus truncatus	4	100.0%	6977	89.7%	800	450	1821	3906
Cazira sp.	3	75.0%	5152	66.2%	800	450		3902
Brachycerocoris camelus	4	100.0%	6974	89.7%	802	450	1821	3901
Megacopta cribraria	4	100.0%	5303	68.2%	802	450	1817	2234
Bannacoris arboreus	4	100.0%	6924	89.0%	802	450	1819	3853
Poecilocoris latus	4	100.0%	6957	89.4%	790	450	1818	3899
Hyperoncus lateritius	4	100.0%	6975	89.7%	802	450	1821	3902
Pycanum ochraceum	4	100.0%	6983	89.8%	802	450	1803	3928
Eurostus ochraceus	4	100.0%	6981	89.8%	802	450	1821	3908
Urolabida histrionica	4	100.0%	6935	89.2%	802	450	1787	3896
Urochela quadrinotata	4	100.0%	6943	89.3%	802	450	1813	3878

Physopelta gutta	4	100.0%	6918	88.9%	802	444	1825	3847
Macroceroea grandis	4	100.0%	6948	89.3%	802	446	1825	3875
Iphita limbata	4	100.0%	6867	88.3%	726	446	1805	3890
Physopelta quadriguttata	4	100.0%	6948	89.3%	802	444	1825	3877
Dysdercus cingulatus	4	100.0%	6908	88.8%	802	450	1818	3838
Antilochus nigripes	4	100.0%	6805	87.5%	664	448	1817	3876
Euscopus fuscus	4	100.0%	6807	87.5%	660	450	1815	3882
Pyrrhocoris sinuaticollis	4	100.0%	6897	88.7%	802	450	1816	3829
Riptortus linearis	4	100.0%	6945	89.3%	802	450	1812	3881
Megalotomus junceus	4	100.0%	6965	89.5%	802	450	1818	3895
Cloreunus modestus	4	100.0%	6951	89.4%	798	450	1825	3878
Homoeocerus striicornis	4	100.0%	6946	89.3%	802	450	1823	3871
Hydaropsis longirostris	4	100.0%	4018	51.7%	802	450	1095	1671
Leptocoris vicinus	4	100.0%	6871	88.3%	738	450	1814	3869
Corizus tetraspilus	4	100.0%	6942	89.3%	802	450	1815	3875
Metatropis brevirostris	4	100.0%	6975	89.7%	802	450	1823	3900
Geocoris grylloides	4	100.0%	6889	88.6%	802	450	1823	3814
Henestaris oschanini	4	100.0%	6701	86.2%	802	450	1825	3624
Dinomachus sikhimensis	4	100.0%	6917	88.9%	802	450	1822	3843
Lygaeus hanseni	4	100.0%	6934	89.1%	802	450	1820	3862
Malcus sinicus	4	100.0%	6078	78.1%	794	448	1795	3041
Elasmolomus squalidus	4	100.0%	6958	89.5%	802	444	1823	3889
Philaenus spumarius	4	100.0%	4867	62.6%	802	444	1787	1834
Cicadetta abscondita	3	75.0%	3000	38.6%	802	450	1748	
Nilaparvata lugens	4	100.0%	4002	51.5%	802	442	1637	1121
Laternaria candelaria	4	100.0%	3745	48.1%	802	440	1339	1164
Geisha distinctissima	3	75.0%	2535	32.6%	802	450	1283	
Xenophyes cascus	4	100.0%	6899	88.7%	802	450	1785	3862
Peloridora minuta	4	100.0%	4080	52.5%	802	450	998	1830
Hemiodoecus leai	3	75.0%	2949	37.9%	802	450		1697
Hackeriella veitchi	4	100.0%	4731	60.8%	802	450	1787	1692

Rhopalosiphum padi	3	75.0%	1872	24.1%	802	444	626	
Camarotoscena sp. YW-2014	2	50.0%	5374	69.1%			1824	3550
Trioza erytreae	2	50.0%	1240	15.9%	802	438		
Adelges tsugae	3	75.0%	1740	22.4%	802	444	494	
